# Thermal cooling performance of convective non-Newtonian nanofluid flowing with variant power-index across moving extending surface

**DOI:** 10.1038/s41598-022-12333-y

**Published:** 2022-05-24

**Authors:** M. Ferdows, MD. Shamshuddin, S. O. Salawu, Shuyu Sun

**Affiliations:** 1 Research Group of Fluid Flow Modeling and Simulation, Department of Applied Mathematics, University of Dhaka, Dhaka, Bangladesh; 2 Department of Mathematics, Vaagdevi College of Engineering, Warangal, Telangana India; 3 Department of Physical Science, Mathematics Programme, College of Pure and Applied Sciences, Landmark University, Omu-aran, Kwara Nigeria; 4 Computational Transport Phenomena Laboratory, Physical Science and Engineering Division, King Abdullah University of Science and Technology, Thuwal 23955-6900, Jeddha, Saudi Arabia

**Keywords:** Engineering, Mathematics and computing, Physics

## Abstract

This communication focuses on assessing the effectiveness of nanoparticles, and a power-law variation fluid on a moving stretching surface is analyzed. Newtonian fluids for different nanomaterials are considered due to its industrial demand. The partial differential equations describing the flow are transformed to ordinary differential equations by employing local similarity transformations and then solved numerically by an effective numerical approach, namely, the local nonsimilarity method (LNS). The numerical solution is computed for different parameters by using the computational software MATLAB. Different types of nanoparticles are considered, and the impact of those nanoparticles as well as the impact of different pertaining parameters on velocity, temperature, missing velocity slope, and missing temperature slope are presented graphically. Comparisons are made with the available results in the open literature. Our investigation conveys a better impact on ${\text{Ag}}$ nanoparticles due to their higher thermal conductivity. Furthermore, an increase in the free stream velocity, missing temperature slope and velocity slope is enhanced, but after a point of separation, the missing temperature slope decays.

## Introduction

Common liquids considered under some conditions by scientists and engineers, such as oils, water and air, can be classified as Newtonian fluids. In several circumstances, Newtonian behavior assumptions are not applicable in the real sense, Raja et al.^1^. However, scientists respond to formulate a non-Newtonian fluid model based on some basic assumptions and fluid behavior. Such liquid arises in the mining industry, plastic and chemical processing and so on. The consideration of mass transportation phenomenon subjected to chemical reaction process has fascinated substantial attention of researchers owing to its valuable utilizations in geothermal engineering, mechano-chemistry, nuclear reactor chilling, material deterioration, water and oil emulsions. A phenomenon that is developed on strong nonlinear ordinary and partial differential equations with viscoelastic properties and has applications in biomedical flows, lubricants, etc.^2–6^. However, not all non-Newtonian liquids exhibit elastic characteristics, as in some cases, elastic properties are insignificant but viscous properties. The flow of viscous liquids in the presence of the heat power law and temperature distribution is important in chemical reaction catalysis, the production of gas and oil, heat conservation and many more, Salawu et al.^7^. A relative variation in the quantity of heat causes proportional variation in the non-Newtonian fluid materials. Hence, non-Newtonian material behavior depends on the temperature variation. Of the several non-Newtonian liquids, nanofluids have strong heat conducting power compared to others, which makes them potentially useful in numerous engineering and industrial heat transfer applications^8–12^.

With heat transfer, nanofluids are uniformly stable synthesized colloid suspensions made of metallic or metal oxides with nanometer-sized particles called nanoparticles. The heat conduction and transfer strength of nanofluids depends on the nanoparticle thermophysical properties, as reported by Salawu and Ogunseye^13^. Buongiorno^14^ provided detailed descriptions of the phenomena of nanoliquid heat transfer and information on what is responsible for the enhanced heat conduction performance of the fluid. Sheikholeslami et al.^15^ considered the effect of Brownian and thermophoresis on a nanofluid steady flow in parallel medium. The study revealed that the heat gradient is enhanced with an increase in viscosity terms but reduces by an increase in the Brownian term. Abbas et al.^16^ studied the theoretical second law of peristaltic nanoliquid flow in a medium with compliant walls. It was reported that nanomaterials increase the heat distribution within wall compliants. Malvandi and Ganji^17^ investigated convective laminar heat transfer with a magnetic field impact on alumina nanoliquids in nanochannels. A high volume fraction was observed for small nanoparticles, which led to steady variation in the heat transfer. Mahmood et al.^18^ studied squeezed flow and the heat transport properties of nanofluid flow in permeable media. A numerical scheme was used for the analysis with reports on the essential flow terms and characteristics. Khan and Pop^19^ examined nanofluid flow in a stretching surface boundary layer with thermophoresis and Brownian influence. The study reported on the effect of Lewis and Prandtl numbers. Due to the industrial and technological value of nanofluids or nanomaterials, several researchers have examined the heat transfer effects of different nanomaterials/fluids^2,3,8,10^.

The significance of the cooling mechanism through heat transfer by convection in nuclear plants, gas turbines, energy thermal storage and others cannot be overemphasized. Convective cooling is important in many industrial and technological processes to manage excessive heat generated to prevent blowup of the system^20–22^. Aziz^23^ considered convective boundary conditions for viscous Blasius flow. The author found that the convective term will assist in controlling the thickness of the boundary layer. Makinde^24^ examined mixed convection for MHD heat transfer fluid in a convective vertical surface. A numerical simulation of the work was performed and reported that increasing the Biot number encourages a heat field. Hayat et al.^25^ investigated convective cooling in a Maxwell fluid with Dufour-Soret effects and chemical reaction. Flow characteristics with the influence of the convective cooling mechanism were presented. Gireesha and Mahanthesh^26^ recently studied the heat transfer of viscoelastic fluid in a convective bounded varying channel. The convective term (Biot number) was reported to have a momentous impact on the non-Newtonian material terms. Makinde and Aziz^27^ addressed stretching surface boundary layer heat convection exchange of nanofluids. A computational solution method was adopted and presented a comprehensive effect of the convective term on the considered nanofluids. However, with numerous studies carried out on nanofluids, detailed descriptions of different nanofluids based on the influence of normalized velocities and volume fractions have not been given.

Khan et al.^28^ presented a comprehensive study of unsteady three-dimensional flow of the Eyring–Powell nanofluid under convective and nanoparticles mass flux conditions. Radiation and stratification phenomenon for thermal analysis of Sutter by nanoliquid are considered khan et al.^29^. Khan et al.^30^ also shown the recent evolution in fluid dynamics where consider of nanoliquids which retains exceptional thermal conductivity characteristics and upsurge heat transportation in fluid. Ikram Ullah^31^ studied the flow of hybrid nanoliquid by an infinite disk where considered hybrid nanoliquid is a combination of AA7072 and AA7075 nanoparticles and water. Analyzed of MHD flow of nanoliquid between two parallel plates in a rotating system considered by Ikram Ullah^32^. Hayat et al.^33^ examined the MHD squeezed flow of second-grade nanofluid between two parallel disks. Awan et al.^34^ analyzed the cumulative effects of both electric and magnetic field on a micropolar nanofluid bounded by two parallel plates in a rotating system. They considered the micropolar nanofluidics flow between parallel plates under the Hall current effect. Qureshi et al.^35^ investigated the impact of radially varying magnetic field during the peristaltically flowing nanofluid in a finite flexible tube. The nonlinear radiative heat transfer effects due to solar radiation in magneto-hydrodynamic (MHD) nanofluidic problem are analyzed by Awan et al.^36^. Raja et al.^37^ investigated the entropy characteristics in magnetohydrodynamics (MHD) nanofluidic flow model by varying surface thickness. Estimation of the effectiveness of Au nanoparticles concentration in peristaltic flow through a curved channel by using a data driven stochastic numerical paradigm based on artificial neural network is presented by Raja et al.^38^. A novel application of an intelligent numerical computing paradigm via artificial neural networks optimized with a Bayesian regularization approach has been presented by Awan et al.^39^ for the investigation of the non-uniform heat absorption process with the bio-convective flow dynamics of nanomaterial involving gyro-tactic microorganisms.

The present theoretical study considers the cooling mechanism of power law variation with heat transfer for $\text{Cu},\;\text{Ag},\;\text{Al}_{2} \text{O}_{3},\;\text{TiO}_{2}$ nanofluids and water-based fluid in a moving stretching surface. Various fascinating results and suggestions as reported by scholars motivated the current analysis, which is significance to the thermal engineering and nanotechnology. Hence, the study will assist in the advancement of technology and for accurate prediction of thermal science devices performance. For a clear understanding of the flow and heat transfer behavior, numerical computation of the modeled nanomaterial is investigated. Detailed discussions of the effect of normalized velocities and volume fraction are presented for flow rate, missing velocity and temperature slopes, heat distribution, and wall effects. The computed results support the existing results and will help encourage the optimal utilization of nanofluids.

## Mathematical formulation

**Figure 1:**
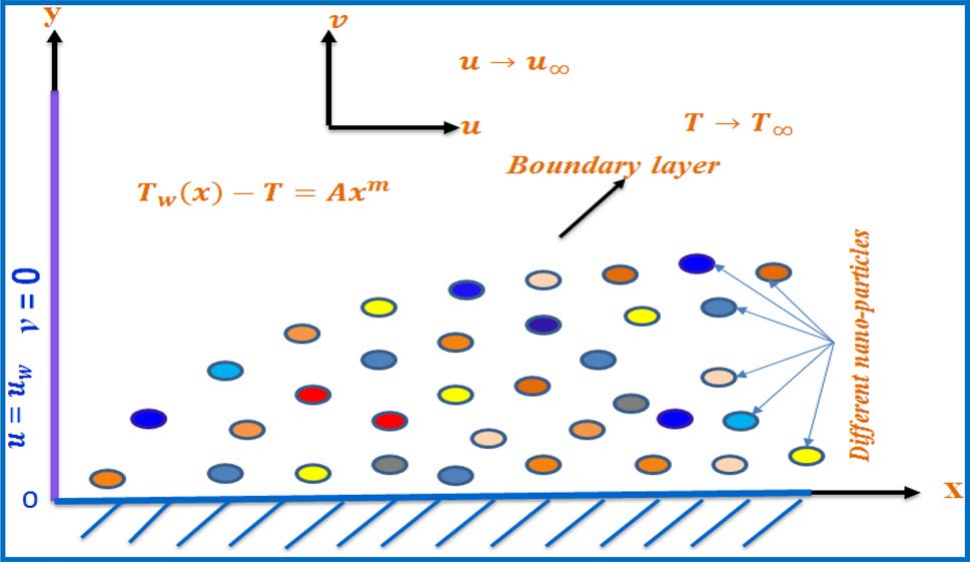
Sketch of the physical flow problem.

Let us consider a steady laminar, incompressible, forced convection flow of a Newtonian nanofluid with velocity $u_{w}$ over the continuous sheet and free stream velocity $U_{\infty }$. Let us assume a power law variation of temperature $T_{w} (x) = T_{\infty } + Ax^{m}$ of the moving sheet. The flow is assumed to be in the x-direction, which is chosen along the plate, and the y-axis is normal to it, Fig. 1. The fluid has density $\rho$, kinematic viscosity $\nu$, thermal conductivity $k_{nf}$, wall temperature exponent $m$, and reference velocity $u_{r}$. All these physical properties of the fluid are considered constant. The wall temperature $T_{w}$ and the free stream temperature $T_{\infty }$ are taken as constants.

Problem formulation:

1 $$ \frac{\partial u}{{\partial x}} + \frac{\partial v}{{\partial y}} = 0, $$

2 $$ u\frac{\partial u}{{\partial x}} + v\frac{\partial u}{{\partial y}} = \nu_{n\;f} \frac{{\partial^{2} u}}{{\partial y^{2} }}, $$

3 $$ u\frac{\partial T}{{\partial x}} + v\frac{\partial T}{{\partial y}} = \frac{{k_{nf} }}{{\rho_{nf} (C_{p} )_{nf} }}\frac{{\partial^{2} T}}{{\partial y^{2} }} $$

The appropriate boundary conditions are

4 $$ \begin{array}{*{20}{l}}
{u = {u_w},\;\;\;v = 0,\;\;\;{T_w}(x) - {T_\infty } = A{x^m}}& \quad {at\;\;\;y = 0}\\
{u \to \infty ,\;T \to {T_\infty }\;}& \quad {as\;\;y \to \infty }
\end{array}$$

Dimensionless dependent and independent variables for nonsimilar solutions are given by

5 $$ \psi (x,y) = x\;u_{r} \;{\text{Re}}^{ - 1/2} \;f\left( {\xi ,\;\eta } \right),\;\;\;\;\eta \left( {x,y} \right) = \frac{y}{x}({\text{Re}} )^{1/2} ,\;\;\xi (x) = \frac{x}{L}{\text{Re}} = \frac{{u_{r} x}}{{\nu_{f} }}\theta = \frac{{T - T_{\infty } }}{{T_{w} - T_{\infty } }} $$

The above equations have dimensional variables that are transformed to dimensionless variables by using the dependent and independent variables given by (5).

Using the above variables into Eqs. (2–3), we obtain following dimensionless equations:

6 $$ \frac{1}{{(1 - \phi )^{2.5} \left\{ {(1 - \phi ) + \phi \frac{{\rho_{s} }}{{\rho_{f} }}} \right\}}}\;f^{\prime\prime\prime}\; + \frac{1}{2}f\;f^{\prime\prime}\; + \xi \left\{ {f^{\prime\prime}\frac{\partial f}{{\partial \xi }} - f^{\prime}\frac{{\partial^{2} f}}{\partial \xi \partial \eta }} \right\} = 0 $$

7 $$ \frac{{\frac{{k_{nf} }}{{k_{f} }}}}{{\left\{ {(1 - \phi ) + \phi \frac{{\left( {\rho c_{p} } \right)_{s} }}{{\left( {\rho c_{p} } \right)_{f} }}} \right\}}}\;\theta^{\prime\prime} + \;\Pr \;\xi \left\{ {\theta^{\prime}\frac{\partial f}{{\partial \xi }} - f^{\prime}\frac{\partial \theta }{{\partial \xi }}} \right\} + \frac{1}{2}\Pr \;\theta^{\prime}\;f - \Pr \;f^{\prime}\;\theta \;m = 0 $$

And the associated boundary conditions:

8 $$ \begin{array}{*{20}{l}}
{f'(\xi ,0) = {U_w},\;\;\;2\xi \frac{{\partial f}}{{\partial \xi }} + f = 0,\;\;\;\theta \left( {\xi ,0} \right) = 1}& \quad {as\;\;\eta  = 0}\\
{f'(\xi ,\eta ) = {U_\infty },\;\;\;\theta \left( {\xi ,\eta } \right) = 0}& \quad {as\;\eta  \to \infty }
\end{array}$$

where prime denotes the differentiation with respect to *η*.

The thermophysical properties of nanofluids assumed in the current study are as presented in Table 1 below following^40,41^

**Table 1:** Thermophysical properties of nanoparticles and base fluid.

Thermo-physical properties	Base fluid	Nanoparticles			
	$\text{H}_{2} \text{O}$	Cu	$\text{Al}_{2} \text{O}_{3}$	$\text{TiO}_{2}$	Ag
$\rho \;(\text{kg}\,\text{m}^{ - 3} )$	997.1	8933	3970	4250	10,500
$c_{p} (\text{J}\,\text{kg}^{ - 1} \;\text{K}^{ - 1} )$	4179	385	765	686.2	235
$k\;(\text{Wm}^{ - 1} \,\text{K}^{ - 1} )$	0.613	400	40	8.954	429
$\beta \times 10^{ - 5} \;(\text{K}^{ - 1} )$	21	1.67	0.85	0.9	1.89
$\sigma \;(\text{Sm}^{ - 1} )$	0.05	5.96 × 10^7^	3.5 × 10^7^	0.26 × 10^7^	6.30 × 10^7^

The local skin friction coefficient (${C}_{f}$) and local Nusselt number (Nu) are the significant quantities for our problem, which are given by

9 $$ C_{f} = 2\frac{{\tau_{w} }}{{\rho \;U_{\infty }^{2} }} = 2\frac{\mu }{{\rho \;U_{r}^{2} }}\left( {\frac{\partial u}{{\partial y}}} \right)_{y = 0} = 2\;{\text{Re}}_{x}^{1/2} \;f^{\prime\prime}\;\,\;\; \Rightarrow C_{f} {\text{Re}}^{1/2} = 2\;f^{\prime\prime}\left( {\xi ,\;0} \right) $$

and

10 $$ Nu = \frac{x}{\Delta T}\left( {\frac{\partial T}{{\partial y}}} \right) = {\text{Re}}^{1/2} \;\theta^{\prime}\left( {\xi ,\,\;0} \right) $$

where $\tau_{w}$ is the shear stress at the wall, $\mu$ is the coefficient of dynamic viscosity, ${\text{Re}}_{x} = U_{\infty } \;x/\nu$ is the local Reynolds number, and $\Delta T = T_{\infty } - T_{w} .$

## Local non-similarity numerical approach

The transformed coupled, nonlinear ordinary differential equations (6)–(7) subject to the boundary conditions are solved numerically by using the local nonsimilarity method ***routine***. The system of equations is of the parabolic type and can be solved by several numeric techniques (local nonsimilarity method). In the system of equations, the boundary conditions are specified at the two ends of the interval. This type of problem is known as a two-point boundary value problem. The default method used here for solving initial value problems (IVPs) in MATLAB is the LNS method. This method has been acquired to solve a sizeable scope of boundary layer flow problems and is perspicuously described by^40,41^. Furthermore, a brief description of the well-orthodox computational scheme evolved by Sparrow and Yu^42^ and Massoudi^43,44^ to solve the above set of ordinary differential equations.

Differentiating the original governing equations with respect to a variable ξ and considering the obtained equations as auxiliary equations combined with original equation. Then considering the variable ξ in this partial differential equation to be a constant so, we can reduce the system as a system of ordinary differential equation.

Take $g(\xi ,\eta ) = \frac{\partial f(\xi ,\eta )}{{\partial \xi }},\,\,\,h(\xi ,\eta ) = \frac{\partial \theta (\xi ,\eta )}{{\partial \xi }}$ in Eqs. (6) and (7) with boundary conditions. Equations are transformed as follows.

11 $$ \frac{1}{{(1 - \phi )^{2.5} \left\{ {(1 - \phi ) + \phi \frac{{\rho_{s} }}{{\rho_{f} }}} \right\}}}\;f^{\prime\prime\prime}\; + \frac{1}{2}f\;f^{\prime\prime}\; + \xi \left\{ {f^{\prime\prime}g - f^{\prime}g^{\prime}} \right\} = 0 $$

12 $$ \frac{{\frac{{k_{nf} }}{{k_{f} }}}}{{\left\{ {(1 - \phi ) + \phi \frac{{\left( {\rho c_{p} } \right)_{s} }}{{\left( {\rho c_{p} } \right)_{f} }}} \right\}}}\;\theta^{\prime\prime} + \;\Pr \;\xi \left\{ {\theta^{\prime}g - f^{\prime}h} \right\} + \frac{1}{2}\Pr \;\theta^{\prime}\;f - \Pr \;f^{\prime}\;\theta \;m = 0 $$

And the associated boundary conditions:

13 $$ \begin{gathered} f^{\prime}(\xi ,0) = U_{w} ,\;\;\;2\xi g\left( {\xi ,0} \right) + f = 0,\;\;\;\theta \left( {\xi ,0} \right) = 1\;\;\;\;\;\;\;\;\;\;\;\;\;\;\;\;\;\;\;\;\;\;\;\;\;\; \hfill \\ f^{\prime}(\xi ,\infty ) = U_{\infty } ,\;\;\;\,\;\;\;\;\;\;\;\;\;\;\;\;\;\theta \left( {\xi ,\infty } \right) = 0\;\;\;\;\;\;\;\;\;\;\;\;\;\;\;\;\;\;\;\;\;\;\;\;\;\;\;\;\;\;\;\;\;\; \hfill \\ \end{gathered} $$

where prime denotes the differentiation with respect to *η*.

Differentiating Eqs. (11)–(12) with boundary conditions with respect to *ξ* we get

14 $$ \frac{1}{{{{(1 - \phi )}^{2.5}}\left\{ {(1 - \phi ) + \phi \frac{{{\rho _s}}}{{{\rho _f}}}} \right\}}}\;g'''\; + \frac{1}{2}gf'' + \frac{1}{2}fg''\; + \left\{ {f''g - f'g'} \right\} = \xi \frac{\partial }{{\partial \xi }}\left\{ {f''g - f'g'} \right\} $$

15 $$ \begin{gathered} \frac{{\frac{{k_{nf} }}{{k_{f} }}}}{{\left\{ {(1 - \phi ) + \phi \frac{{\left( {\rho c_{p} } \right)_{s} }}{{\left( {\rho c_{p} } \right)_{f} }}} \right\}}}\;h^{\prime\prime} + \;\Pr \;\left\{ {\theta^{\prime}g - f^{\prime}h} \right\} + \frac{1}{2}\Pr \;\theta^{\prime}\;g + \frac{1}{2}\Pr \;h^{\prime}f - \Pr \;g^{\prime}\;\theta \;m - \Pr \;f^{\prime}h\;m \hfill \\ = \Pr \;\xi \frac{\partial }{\partial \xi }\left\{ {\theta^{\prime}g - f^{\prime}h} \right\} \hfill \\ \end{gathered} $$

16 $$ \begin{aligned} & f^{\prime}(\xi ,0) = U_{w} ,\;\;\;2\xi f\left( {\xi ,0} \right) + f = 0,\;\;\;\theta \left( {\xi ,0} \right) = 1,\;f^{\prime}(\xi ,\infty ) = U_{\infty } ,\;\;\theta \left( {\xi ,\infty } \right) = 0\;\; \hfill \\ & g(\xi ,0) = U_{w} ,\;\;\;2\xi g\left( {\xi ,0} \right) + f = 0,\;\;\;h\left( {\xi ,0} \right) = 1,\;g^{\prime}(\xi ,\infty ) = U_{\infty } ,\;\;h\left( {\xi ,\infty } \right) = 0\;\;\;\;\;\;\;\;\;\;\;\;\;\;\; \hfill \\ \;\;\;\;\;\;\;\;\;\;\;\;\;\;\;\;\;\;\;\;\;\;\;\;\;\;\;\;\;\;\;\;\; \hfill \\ \end{aligned} $$

Equations (14)–(16) are auxiliary equations to the governing equations (11)–(13) with their boundary conditions in Eq. (16). Now, deleting the terms from the auxiliary Eqs. (14)–(15) containing the differentiation with respect to stream-wise co-ordinate from the right- hand side of equations as discussed by Sparrow and Yu^42^. With the above assumption, the momentum and energy boundary-layer equations (11)–(12) and its auxiliary equations (14)–(15) could be brought together with their boundary conditions as

17 $$ \frac{1}{{(1 - \phi )^{2.5} \left\{ {(1 - \phi ) + \phi \frac{{\rho_{s} }}{{\rho_{f} }}} \right\}}}\;f^{\prime\prime\prime}\; + \frac{1}{2}f\;f^{\prime\prime}\; + \xi \left\{ {f^{\prime\prime}\frac{\partial f}{{\partial \xi }} - f^{\prime}\frac{{\partial^{2} f}}{\partial \xi \partial \eta }} \right\} = 0 $$

18 $$ \frac{{\frac{{k_{nf} }}{{k_{f} }}}}{{\left\{ {(1 - \phi ) + \phi \frac{{\left( {\rho c_{p} } \right)_{s} }}{{\left( {\rho c_{p} } \right)_{f} }}} \right\}}}\;\theta^{\prime\prime} + \;\Pr \;\xi \left\{ {\theta^{\prime}\frac{\partial f}{{\partial \xi }} - f^{\prime}\frac{\partial \theta }{{\partial \xi }}} \right\} + \frac{1}{2}\Pr \;\theta^{\prime}\;f - \Pr \;f^{\prime}\;\theta \;m = 0 $$

19 $$\frac{1}{{{{(1 - \phi )}^{2.5}}\left\{ {(1 - \phi ) + \phi \frac{{{\rho _s}}}{{{\rho _f}}}} \right\}}}\;g'''\; + \frac{1}{2}gf'' + \frac{1}{2}fg''\; + \left\{ {f''g - f'g'} \right\} = \xi \frac{\partial }{{\partial \xi }}\left\{ {f''g - f'g'} \right\}$$

20 $$ \begin{gathered} \frac{{\frac{{k_{nf} }}{{k_{f} }}}}{{\left\{ {(1 - \phi ) + \phi \frac{{\left( {\rho c_{p} } \right)_{s} }}{{\left( {\rho c_{p} } \right)_{f} }}} \right\}}}\;h^{\prime\prime} + \;\Pr \;\left\{ {\theta^{\prime}g - f^{\prime}h} \right\} + \frac{1}{2}\Pr \;\theta^{\prime}\;g + \frac{1}{2}\Pr \;h^{\prime}f - \Pr \;g^{\prime}\;\theta \;m - \Pr \;f^{\prime}h\;m \hfill \\ = \Pr \;\xi \frac{\partial }{\partial \xi }\left\{ {\theta^{\prime}g - f^{\prime}h} \right\} \hfill \\ \end{gathered} $$

With

21 $$ \begin{aligned} & f^{\prime}(\xi ,0) = U_{w} ,\;\;\;2\xi f\left( {\xi ,0} \right) + f = 0,\;\;\;\theta \left( {\xi ,0} \right) = 1,\;f^{\prime}(\xi ,\infty ) = U_{\infty } ,\;\;\theta \left( {\xi ,\infty } \right) = 0\;\; \hfill \\&  g(\xi ,0) = U_{w} ,\;\;\;2\xi g\left( {\xi ,0} \right) + f = 0,\;\;\;h\left( {\xi ,0} \right) = 1,\;g^{\prime}(\xi ,\infty ) = U_{\infty } ,\;\;h\left( {\xi ,\infty } \right) = 0\;\;\;\;\;\;\;\;\;\;\;\;\;\;\; \hfill \\ \;\;\;\;\;\;\;\;\;\;\;\;\;\;\;\;\;\;\;\;\;\;\;\;\;\;\;\;\;\;\;\;\; \hfill \\ \end{aligned} $$

By considering ξ as a constant parameter, Eqs. (17) to (20) may be treated as a system of ordinary differential equations.

## Results and discussion

The set of nonlinear ordinary differential equations satisfying the boundary conditions has been solved numerically for several values of the parameters involved taking $\text{Cu},\;\text{Ag},\;\text{Al}_{2} \text{O}_{3} ,\;\text{TiO}_{2}$ nanoparticles with base fluid water. For a clear understanding of the flow and heat transfer characteristics of nanofluids, in this section, the effects of the volume fraction of nanofluids, Prandtl number, free stream velocity, and surface velocity on fluid flows are displayed. Two cases, $U_{\infty } < U_{w}$ and $U_{\infty } > U_{w}$, are considered to study the behavior of the Nusselt number and shearing stress. All the behavior of fluid has been investigated for $U_{\infty } < U_{w}$. Since $U_{\infty }$ and $U_{w}$ are the boundary conditions of velocity, they have less impact on temperature profiles.

In order to assess the validity and accuracy of the numerical results, we computed the skin friction ${f}^{{{\prime}}{{\prime}}}\left(0\right)$ and local Nusselt number ${\theta }^{{\prime}}\left(0\right)$ for several values of Pr when $\varphi =0, \xi =0, m=0, {U}_{w}=1, {U}_{\infty }=0$ are fixed and compared them with Bachok et al.^45^ in Table 2 and observed that our results were in excellent agreement with those of Bachok et al.^45^. Therefore, we conclude that our observation method was accurate in light of this comparison.

**Table 2:** Variations of the skin friction ${f}^{{{\prime}}{{\prime}}}\left(0\right)$ and local Nusselt number ${\theta }^{{\prime}}\left(0\right)$ for several sets of physical parameters Pr when $\varphi =0, \xi =0, m=0, {U}_{w}=1, {U}_{\infty }=0$ are fixed.

Pr	Bachok et al. ^45^		Present results	
	− $f{^{\prime}}{^{\prime}}(0)$	$-{\theta }^{^{\prime}}\left(0\right)$	− $f{^{\prime}}{^{\prime}}(0)$	$-{\theta }^{{\prime}}\left(0\right)$
0.7	0.4437	0.3492	0.443748	0.349236
1	0.4437	0.4437	0.443748	0.443748
10	0.4437	1.6803	0.443748	1.68029

**Figure 2:**
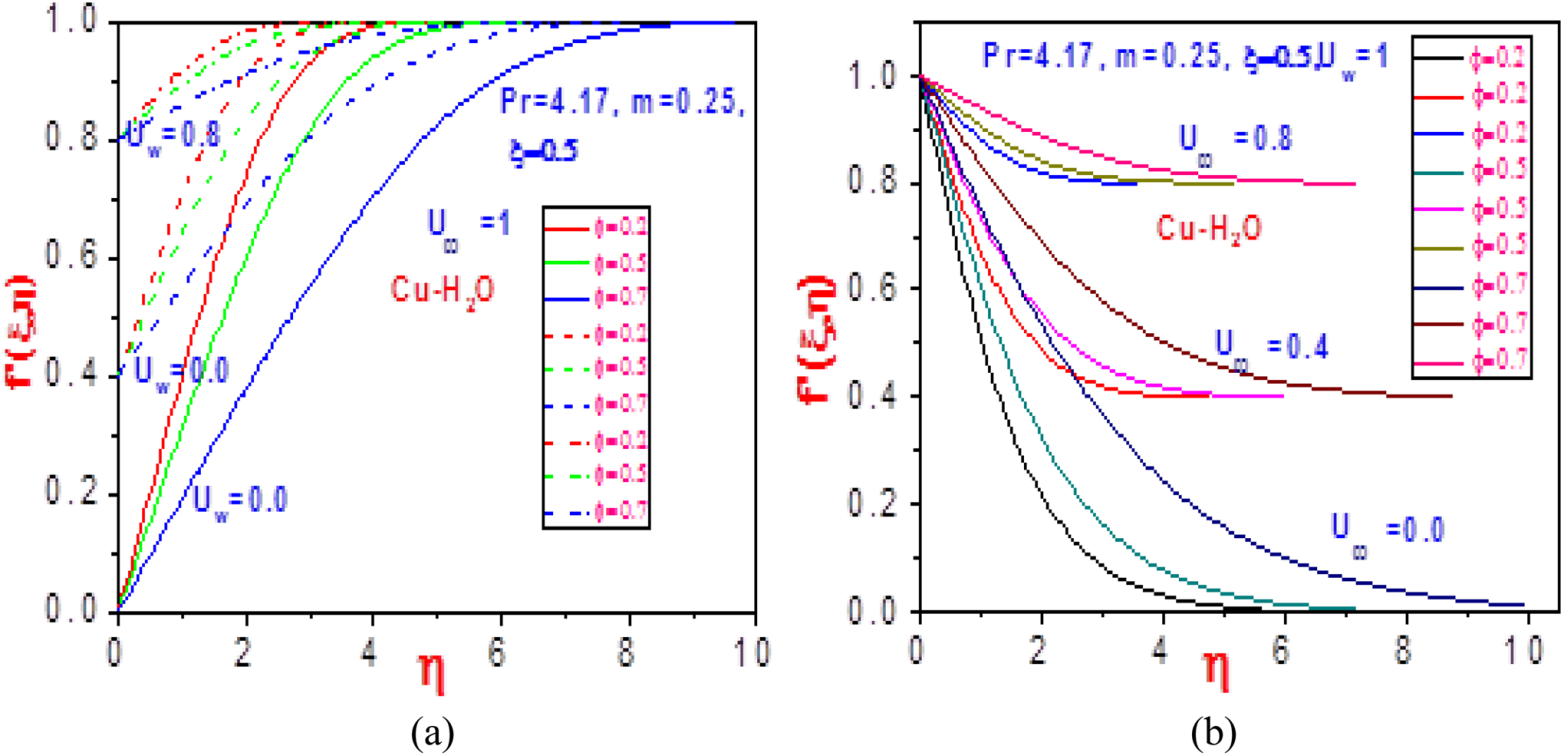
Profiles of velocity $f^{\prime}\left( {\xi ,\eta } \right)$ for (**a**) different values of $U_{\infty }$ with $U_{w} = 1$ and (**b**) different values of $U_{w}$ with $U_{\infty } = 1$.

**Figure 3:**
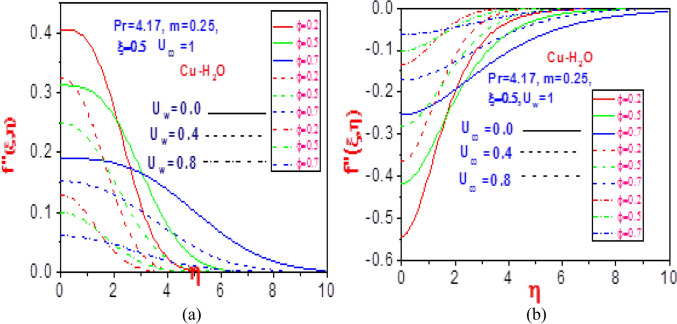
Profiles of velocity $f^{\prime}\left( {\xi ,\eta } \right)$ for (**a**) different values of $U_{\infty }$ with $U_{w} = 1$ and (**b**) different values of $U_{w}$ with $U_{\infty } = 1$.

The plots of the velocity field in the $\left( {\eta ,f^{\prime}(\xi ,\eta )} \right)$ and $\left( {\eta ,f^{\prime}{\prime}(\xi ,\eta )} \right)$ planes are presented in Figs. 2 and 3, respectively, for copper water nanoliquids. With variation in the solid volume fraction $\varphi$, there is a total increase in the magnitude of the copper nanoliquid flow rate with rising values of normalized velocity $U_{w}$ or free stream velocity $U_{\infty }$ as seen in Fig. 2. This is due to the lower motion of the plate that stimulates heat in the system, thereby leading to a momentous nanofluid velocity distribution. The reaction of the velocity gradient to an increasing volume fraction and unvarying normalized stream velocity is depicted in Fig. 3a. A significant shrinkage in the profile is revealed due to high heat loss to the surroundings that in turn enhances the non-Newtonian strength of the fluid. Meanwhile, a contrast behavior is obtained in Fig. 3b for rising free stream velocity and fixed normalized velocity. A rise in the volume fraction emboldens a momentum boundary layer that reduces heat diffusion, which results in a rise in the temperature of the system that causes free flow of nanoparticles. Therefore, the gradient velocity field increases.

**Figure 4:**
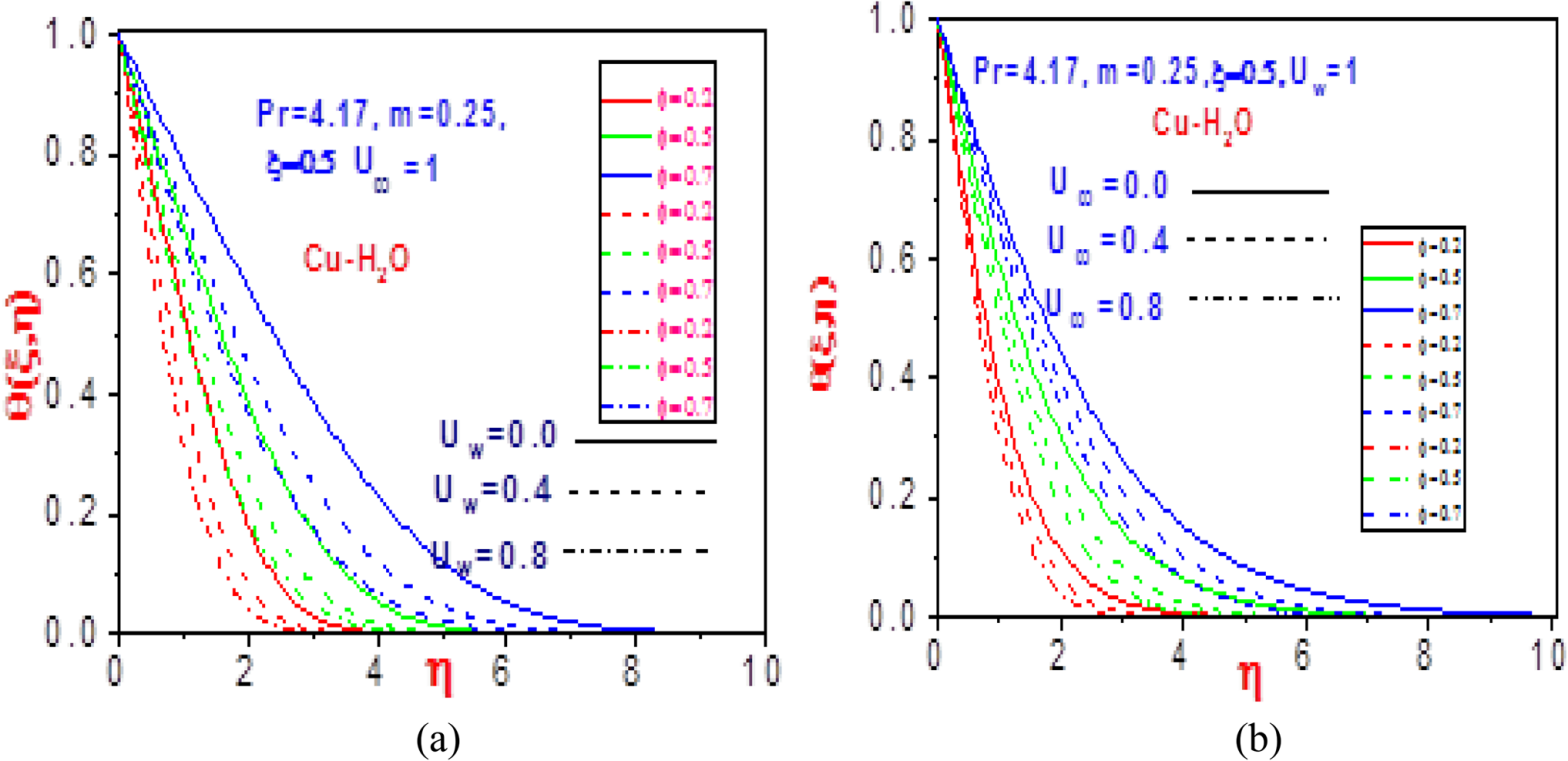
Profiles of temperature $\theta \left( {\xi ,\eta } \right)$ for (**a**) different values of $U_{\infty }$ with $U_{w} = 1$ and (**b**) different values of $U_{w}$ with $U_{\infty } = 1$.

**Figure 5:**
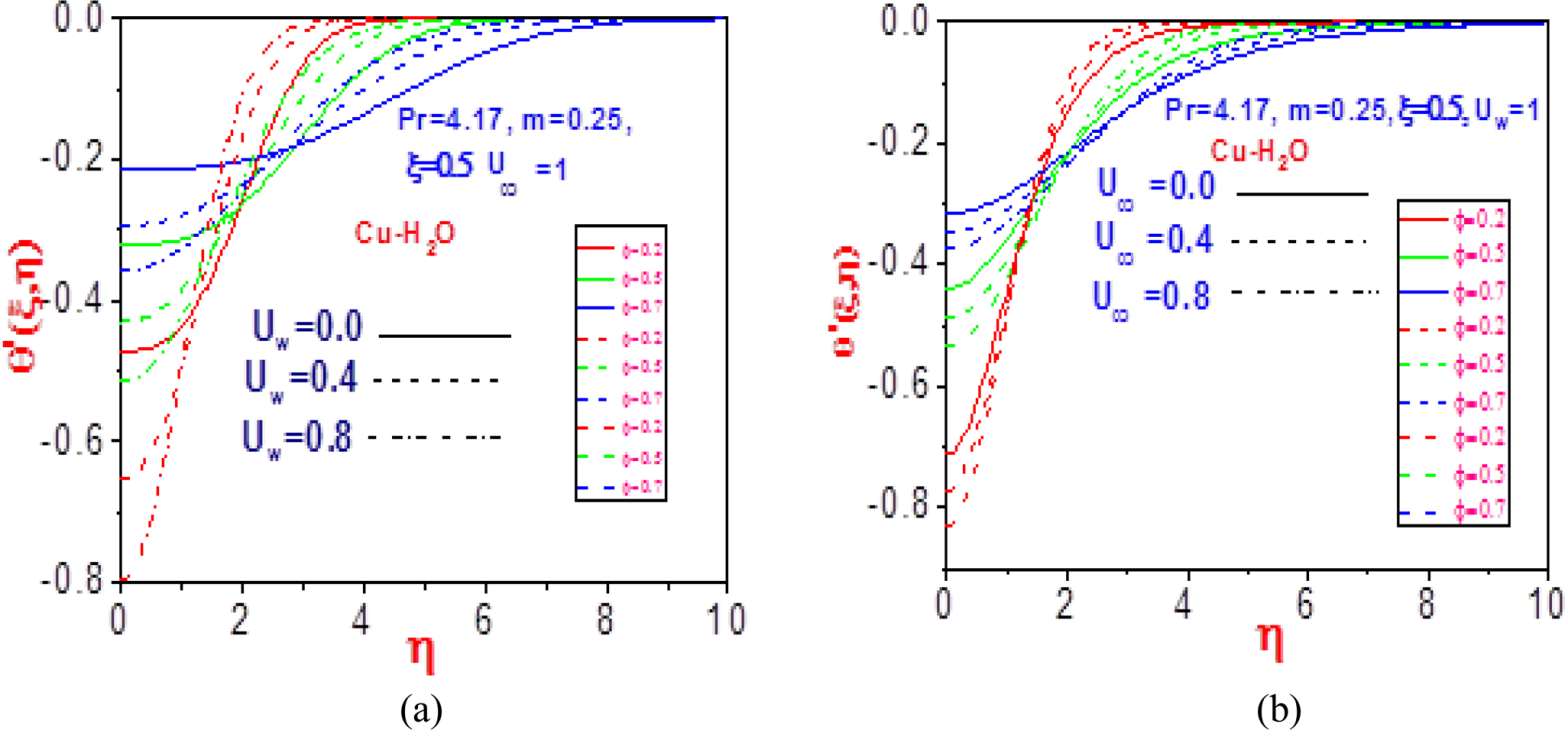
Profiles of missing temperature slope $\theta^{\prime}\left( {\xi ,\eta } \right)$ for (**a**) different values of $U_{\infty }$ with $U_{w} = 1$ and (**b**) different values of $U_{w}$ with $U_{\infty } = 1$.

The heat distributions for various values of volume fraction $\varphi$ with fixed normalized velocity $U_{w}$ or $U_{\infty }$ are demonstrated in Figs. 4 and 5 for the copper nanomaterial. The thermodynamic temperature is discouraged as the volume fraction increases for variation in the normalized velocities, as presented in Fig. 4. This is because of the increase in the ratio of constituent volume to the volume of all constituent copper nanomaterial mixtures, which creates an additive and diminishes the energy distribution. The copper nanofluid temperature gradient is confirmed for the impact of the volume fraction in Fig. 5. Close to the plate, the normalized velocity effects are observed to have early decreased the missing energy slope field but rises steadily as it moves distance from the moving plate surface. This revealed the influence of the heat power law on the energy boundary layer, which decreases and increases with time variation.

**Figure 6:**
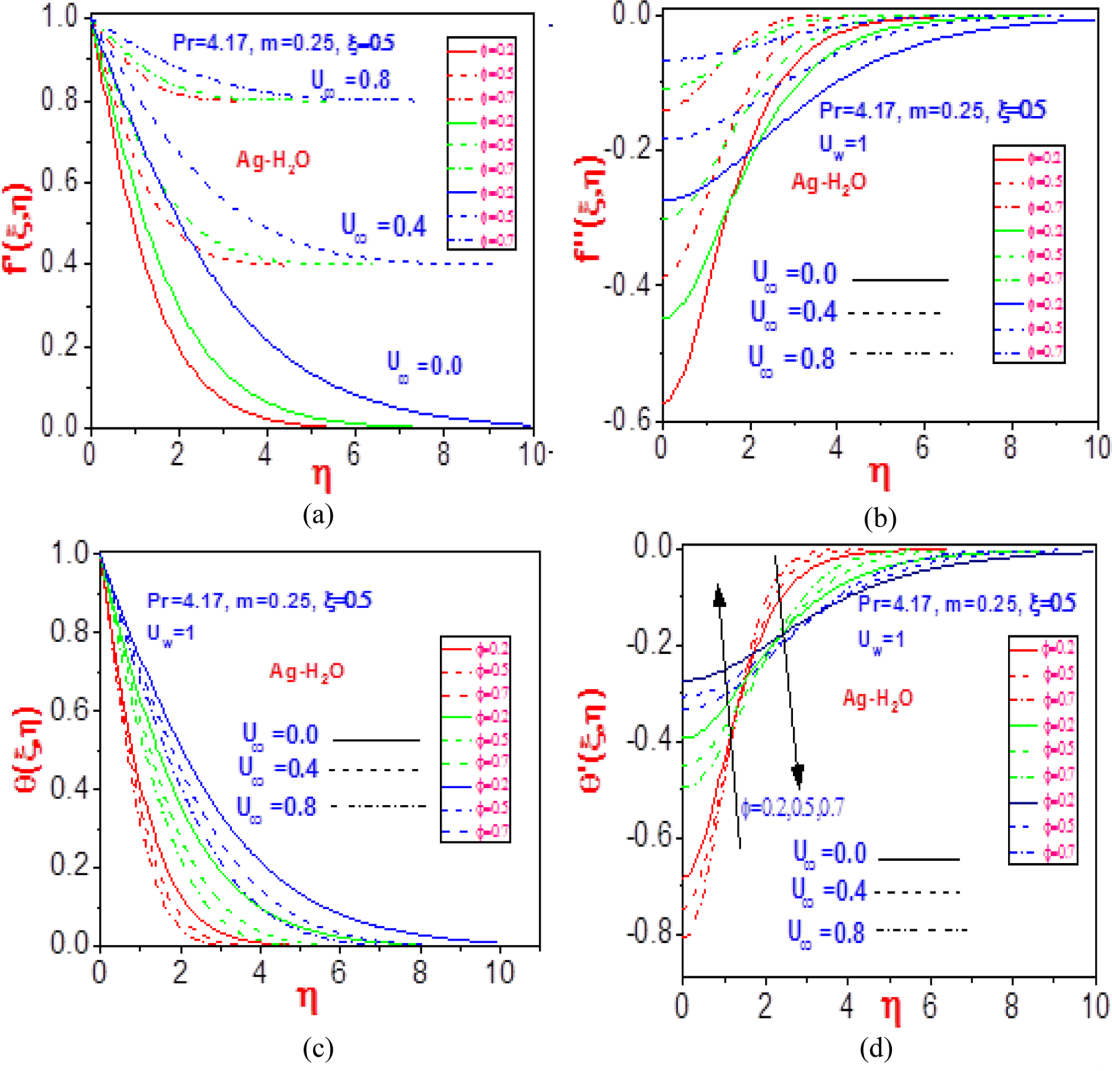
Profiles of (**a**) velocity $f^{\prime}\left( {\xi ,\eta } \right)$, (**b**) missing velocity slope $f^{\prime\prime}\left( {\xi ,\eta } \right)$, (**c**) temperature $\theta \left( {\xi ,\eta } \right)$, and (**d**) missing temperature slope $\theta^{\prime}\left( {\xi ,\eta } \right)$ with different values of $U_{\infty }$ and $\phi$ for Ag-H_2_O.

Figure 6 profiles denote the influence of volume fraction with variation in the free velocity for flow fields and heat profiles. Raising the values of free normalized velocity greatly impacts the velocity distribution and missing velocity slope field, as shown in Fig. 6a,b. The additive mixtures for the constituent silver water material decrease due to the fluid thermotemperature that dampens the bonding material force, which then leads to significant rises in the mixture flow rate and rate of missing material slope. The temperature profiles shrink for different values of $\varphi$ with rising free velocity, as noticed in Fig. 6c,d. An overall decline in the energy transfer and the missing slope are observed due to an increase in the dispersion of heat to the environment as a result of thinner power law boundary layers.

**Figure 7:**
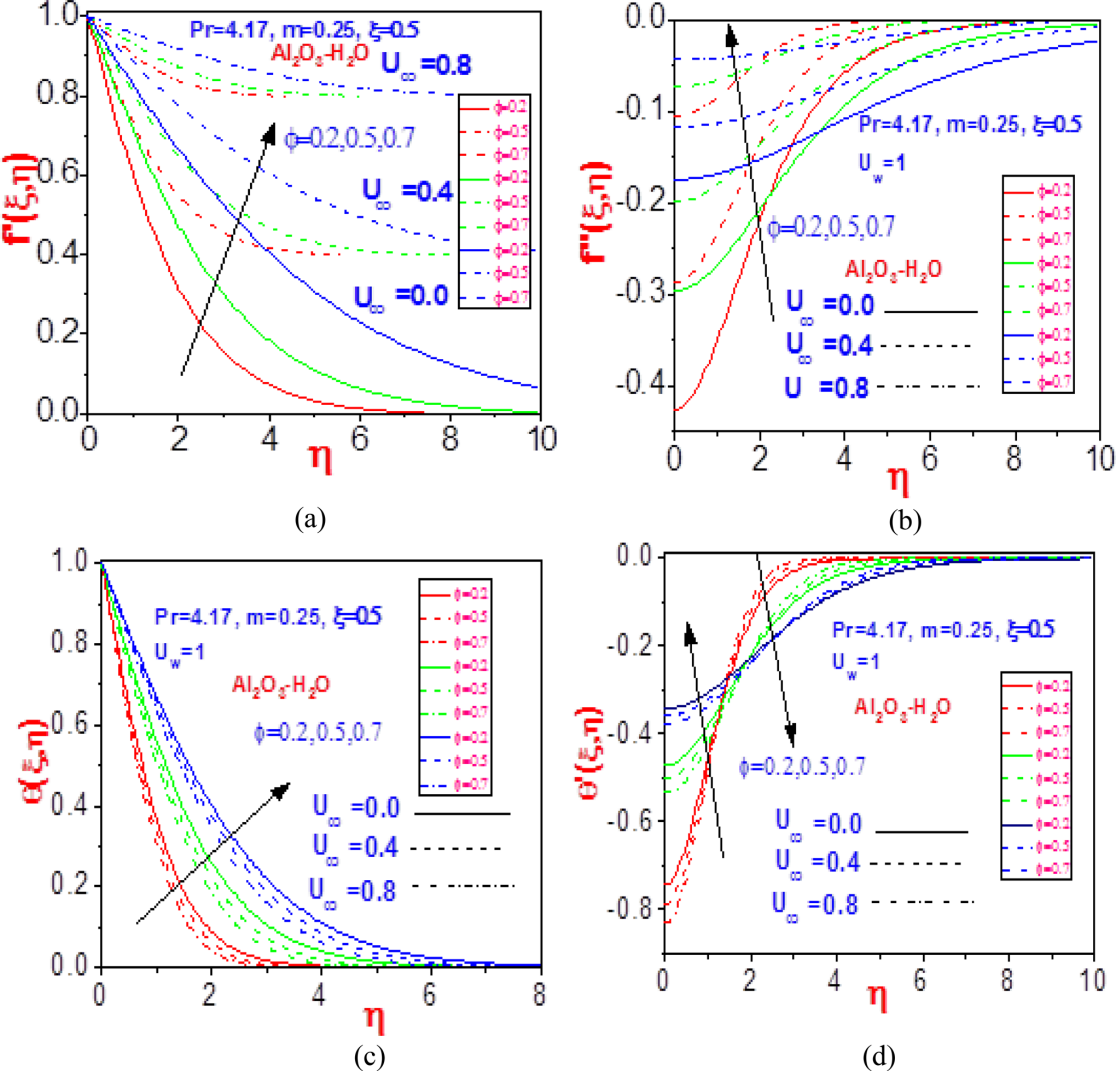
Profiles of (**a**) velocity $f^{\prime}\left( {\xi ,\eta } \right)$, (**b**) missing velocity slope $f^{\prime\prime}\left( {\xi ,\eta } \right)$, (**c**) temperature $\theta \left( {\xi ,\eta } \right)$, and (**d**) missing temperature slope $\theta^{\prime}\left( {\xi ,\eta } \right)$ with different values of $U_{\infty }$ and $\phi$ for Al_2_O_3_-H_2_O.

**Figure 8:**
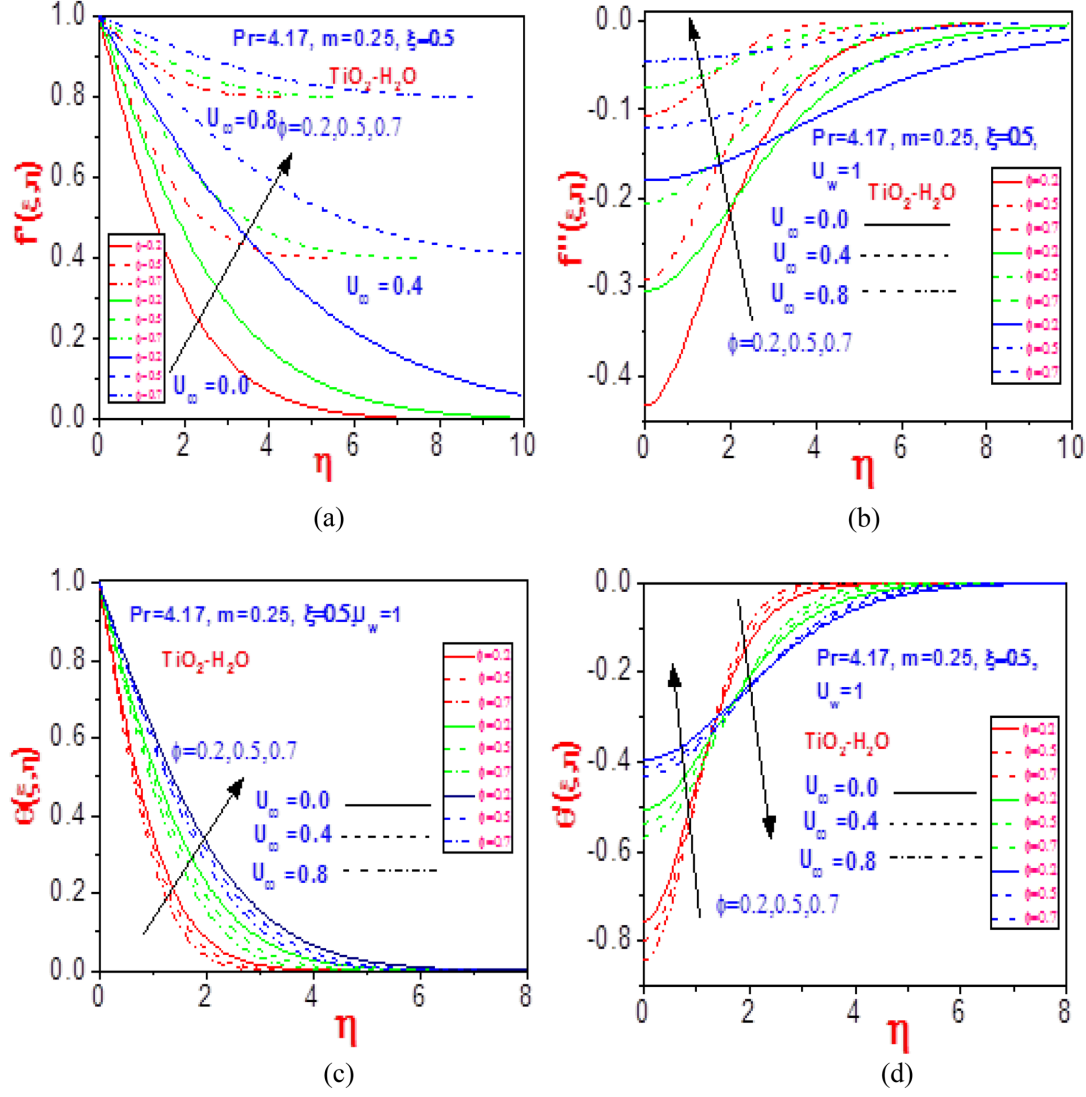
Profiles of (**a**) velocity $f^{\prime}\left( {\xi ,\eta } \right)$, (**b**) missing velocity slope $f^{\prime\prime}\left( {\xi ,\eta } \right)$, (**c**) temperature $\theta \left( {\xi ,\eta } \right)$, and (**d**) missing temperature slope $\theta^{\prime}\left( {\xi ,\eta } \right)$ with different values of $U_{\infty }$ and $\phi$ for Ti_2_O_2_-H_2_O.

Figure 7 represents the response of aluminum oxide material fluid velocity and heat dispersion to distinct values of free normalized velocity at different volume fractions. The same order of behavior is witnessed, as noticed in Fig. 6, but with a lower significance due to the high conducting rate of silver water than aluminum oxide nanofluid. The constituent volume mixture indeed influenced the flow rate and heat transfer field. The change in volume fraction along with the variation in the free velocity $U_{\infty }$ is presented in Fig. 8 for the flow momentum and temperature profiles. The volume fraction impacts the non-Newtonian fluid velocity and missing slope, as well as the energy profile and missing heat gradient slope. The noteworthy effect depends on the strength of the mixed component of the nanowater. Hence, increasing the constituent volume mixture raises the velocity and the heat profile, as given in the plots.

**Figure 9:**
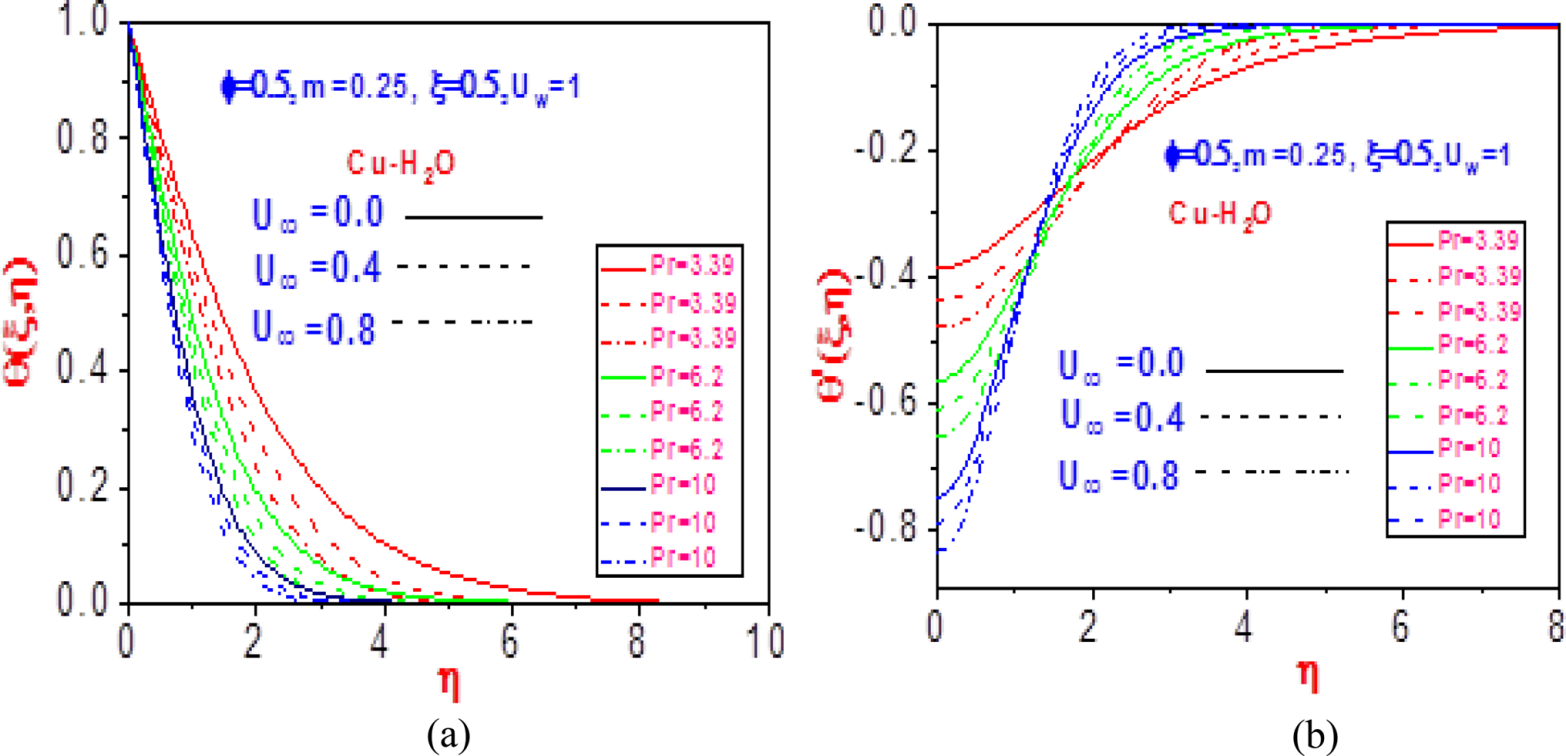
Profiles of (**a**) temperature $\theta \left( {\xi ,\eta } \right)$ and (**b**) missing temperature slope $\theta^{\prime}\left( {\xi ,\eta } \right)$ with different values of $U_{\infty }$ and $\Pr$ for Cu-H_2_O.

**Figure 10:**
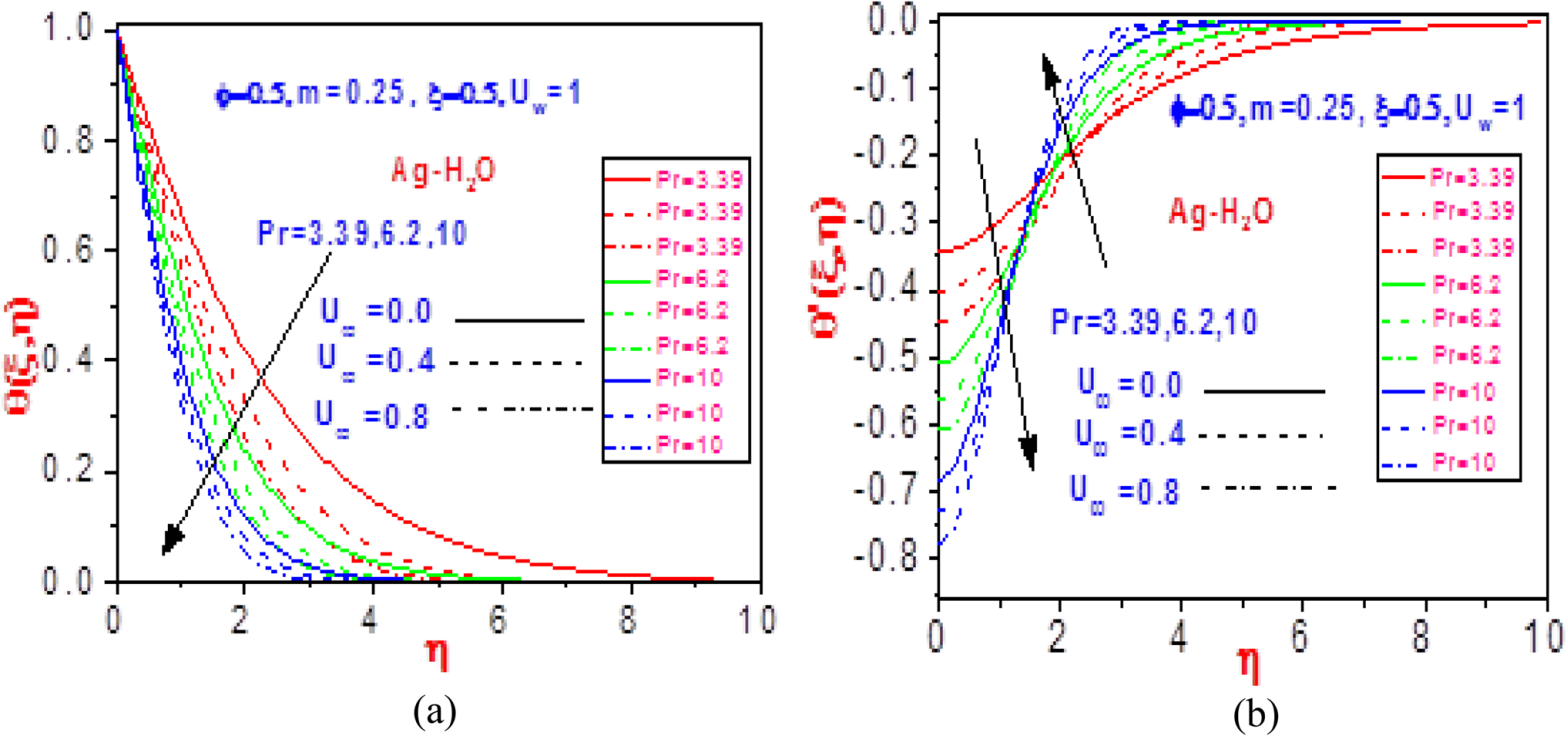
Profiles of (**a**) temperature $\theta \left( {\xi ,\eta } \right)$ and (**b**) missing temperature slope $\theta^{\prime}\left( {\xi ,\eta } \right)$ with different values of $U_{\infty }$ and $\Pr$ for Ag-H_2_O.

**Figure 11:**
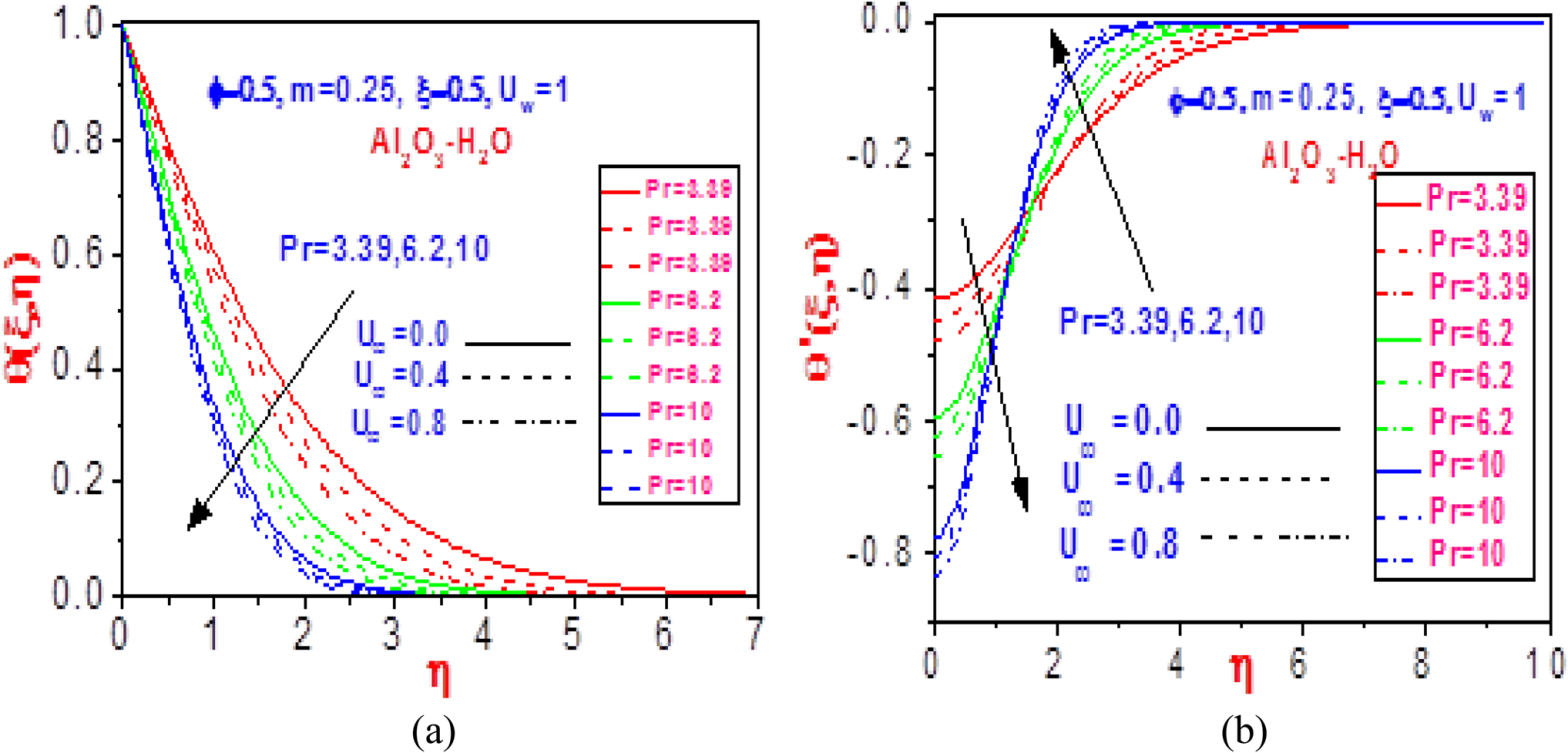
Profiles of (**a**) temperature $\theta \left( {\xi ,\eta } \right)$ and (**b**) missing temperature slope $\theta^{\prime}\left( {\xi ,\eta } \right)$ with different values of $U_{\infty }$ and $\Pr$ for Al_2_O_3_-H_2_O.

**Figure 12:**
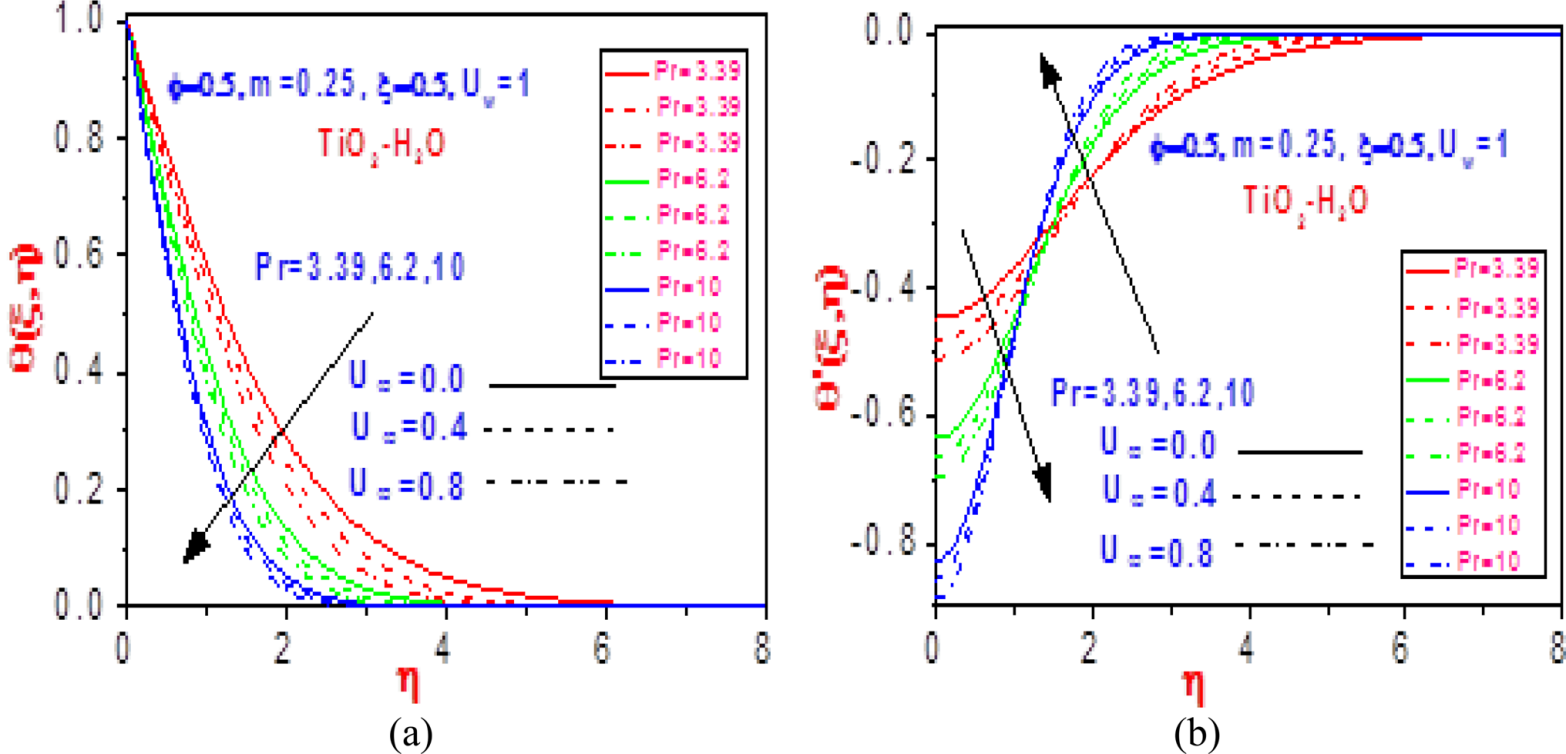
Profiles of (**a**) temperature $\theta \left( {\xi ,\eta } \right)$ and (**b**) missing temperature slope $\theta^{\prime}\left( {\xi ,\eta } \right)$ with different values of $U_{\infty }$ and $\Pr$ for TiO_2_-H_2_O.

Figures 9, 10, 11, and 12 show the effect of raising the values of free velocity $U_{\infty }$ relative to changes in the Prandtl number $\Pr$ on the temperature and missing heat slope. As seen in Figs. 9a, 10a, 11a and 12a, with variation in the ratio of flow momentum to heat diffusivity, the temperature distribution component diminishes for all considered nanowater. The decrease is due to huge heat lost out of the system to the surroundings as the thermal layer becomes thinner. As a result, no internal heat generation term is encouraged in the nanofluid. Additionally, Figs. 9b, 10b, 11b and 12b reveal the heat missing gradient profiles to change in the free velocity for various Prandtl numbers. Near the motioning plate, there is a decrease in the missing heat gradient for the examined nanomaterials, but with distance from the moving surface, the temperature slope increases. The contraction and expansion of the boundary wall resulted in temperature behavior, and the change in temperature is useful to climatology and meteorology in the prediction of atmospheric appearance.

**Figure 13:**
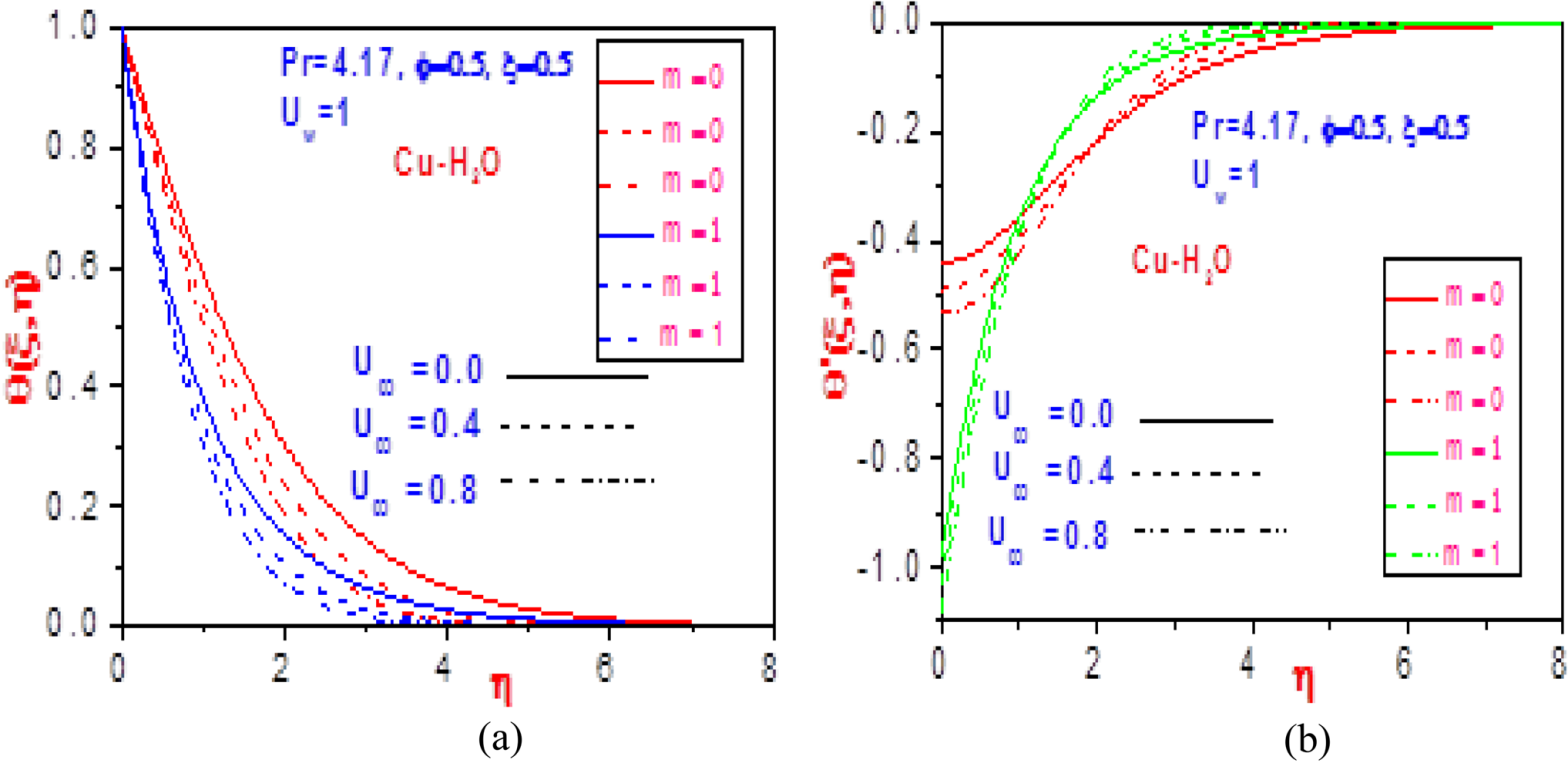
Profiles of (**a**) temperature $\theta \left( {\xi ,\eta } \right)$ and (**b**) missing temperature slope $\theta^{\prime}\left( {\xi ,\eta } \right)$ with different values of $U_{\infty }$ and $m$ for Cu-H_2_O.

**Figure 14:**
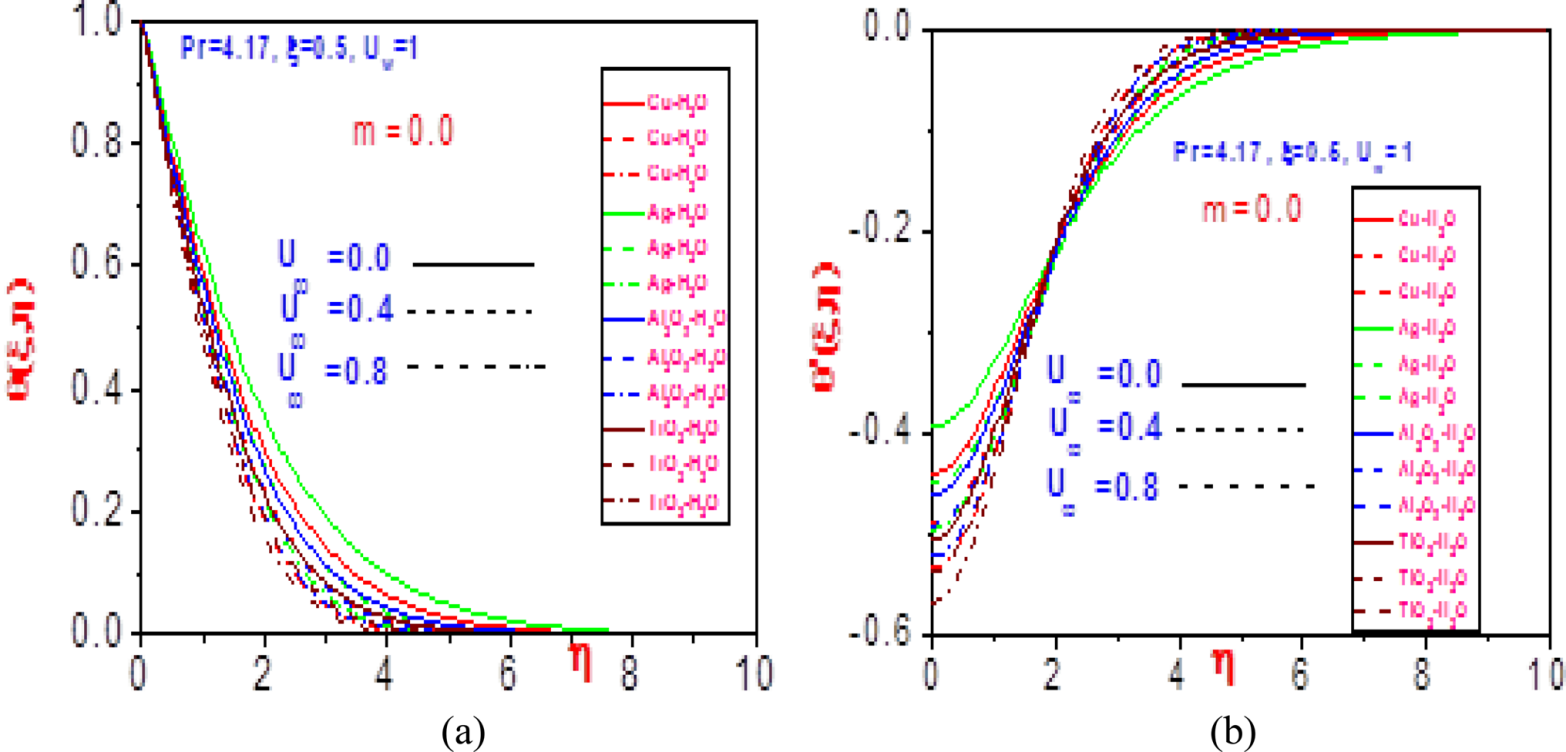
Profiles of (**a**) temperature $\theta \left( {\xi ,\eta } \right)$, (**b**) missing temperature slope $\theta^{\prime}\left( {\xi ,\eta } \right)$ with different values of $U_{\infty }$ and $m = 0$ for Cu-H_2_O, Ag-H_2_O, Al_2_O_3_-H_2_O and TiO_2_-H_2_O.

**Figure 15:**
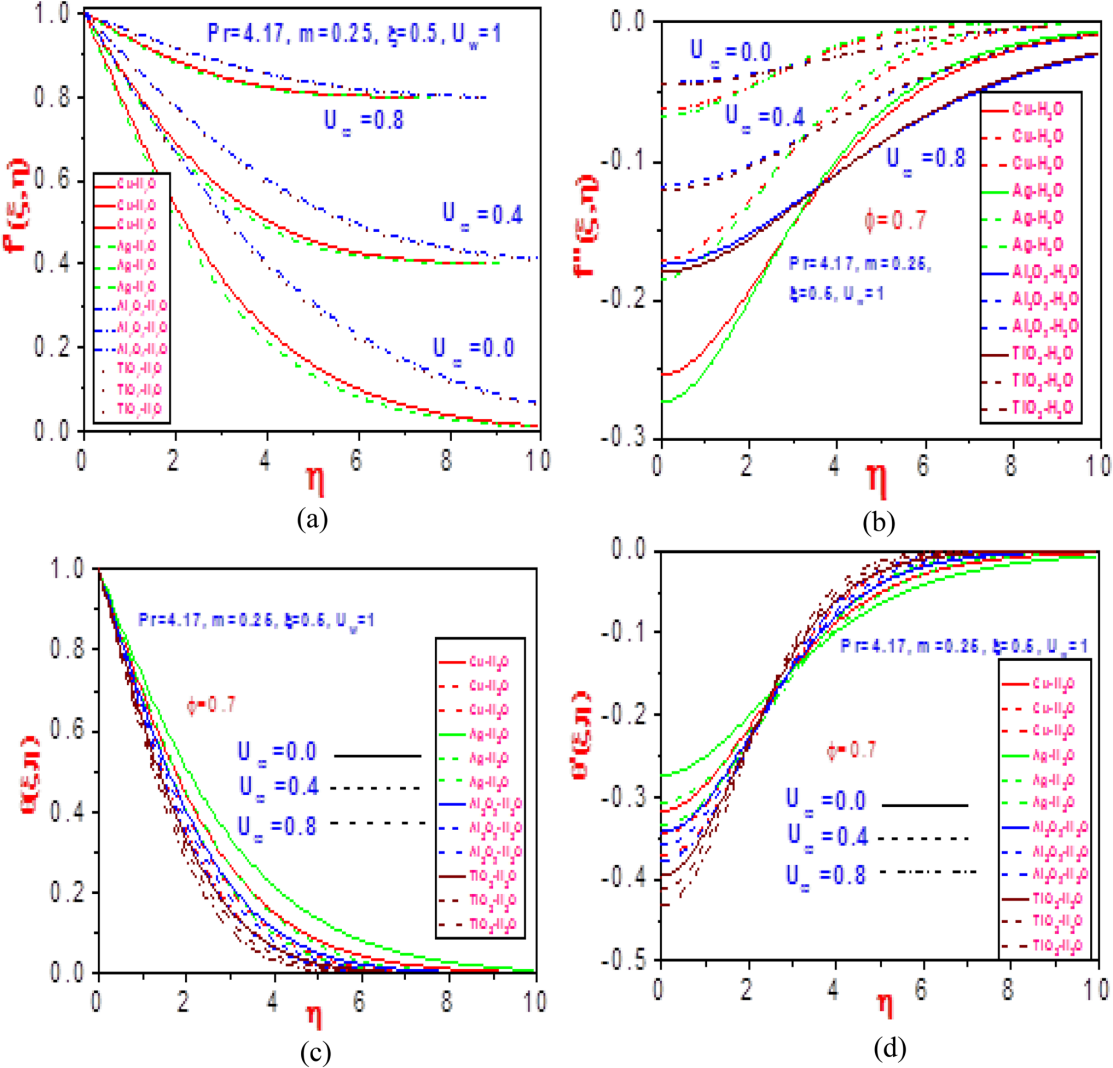
Profiles of (**a**) velocity $f^{\prime}(\xi ,\eta )$, (**b**) missing velocity slope $f^{\prime\prime}(\xi ,\eta )$, (**c**) temperature $\theta (\xi ,\eta )$ and (d) missing temperature slope $\theta^{\prime}(\xi ,\eta )$ with different values of $U_{\infty }$ and $\phi = 0.7$ for Cu-H_2_O, Ag-H_2_O, Al_2_O_3_-H_2_O and TiO_2_-H_2_O.

**Figure 16:**
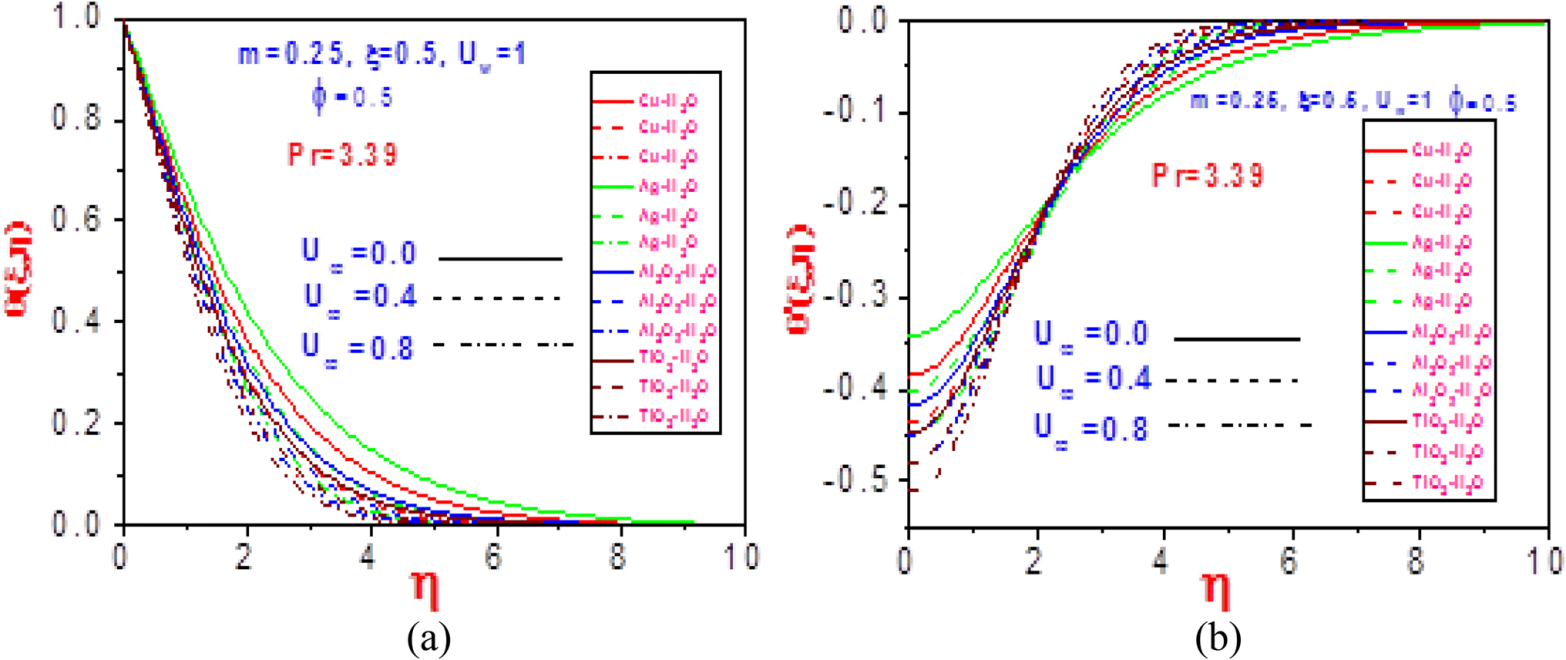
Profiles of (**a**) temperature $\theta (\xi ,\eta )$ and (**b**) missing temperature slope $\theta^{\prime}(\xi ,\eta )$ with different values of $U_{\infty }$ and $\Pr = 3.39$ for Cu-H_2_O, Ag-H_2_O, Al_2_O_3_-H_2_O and TiO_2_-H_2_O.

The wall temperature impact on the copper nanowater for various free normalized velocity values is presented in Fig. 13. The heat profile decreases while the heat missing gradient field initially reduces but later rises far away from the motioning plate. This is because of a change in the velocity of the plate relative to changes in the free normalized velocity. The respective nanowater velocity profiles, energy distributions and missing slopes for various nanomaterials presented in Figs. 14, 15 and 16 tolled the same pattern as earlier discussed cases with variation in the free velocity. A corresponding decrease in the flow velocity magnitude and overall components of the temperature profiles is observed as the free velocity rises for the nanowater. However, the significant effects are not the same as earlier described cases due to the different influences of the thermophysical properties. Likewise, the missing slope for velocity and temperature aligned with the previously discussed cases with different impacts. Early decrease in the missing slopes which later raised as the thermophysical terms vary. The momentous effect depends on the thermal conductivity strength of each nanowater.

**Figure 17:**
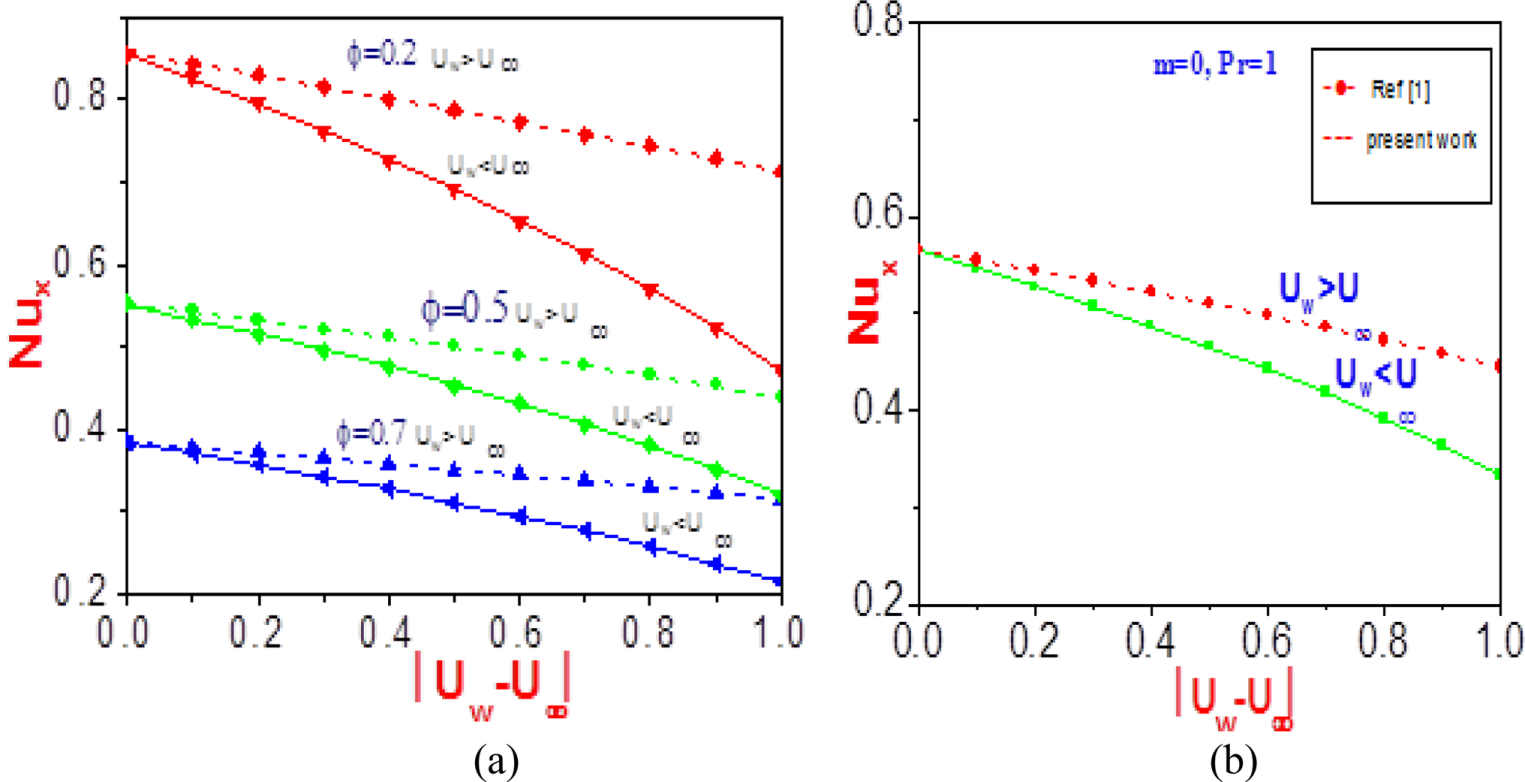
Profiles of Nusselt number versus normalized velocity difference (**a**) at different values of $\phi$ near the wall $(\eta = 0)$ (**b**) for $n = 1$ and without nanoparticles [Ref.^46^].

**Figure 18:**
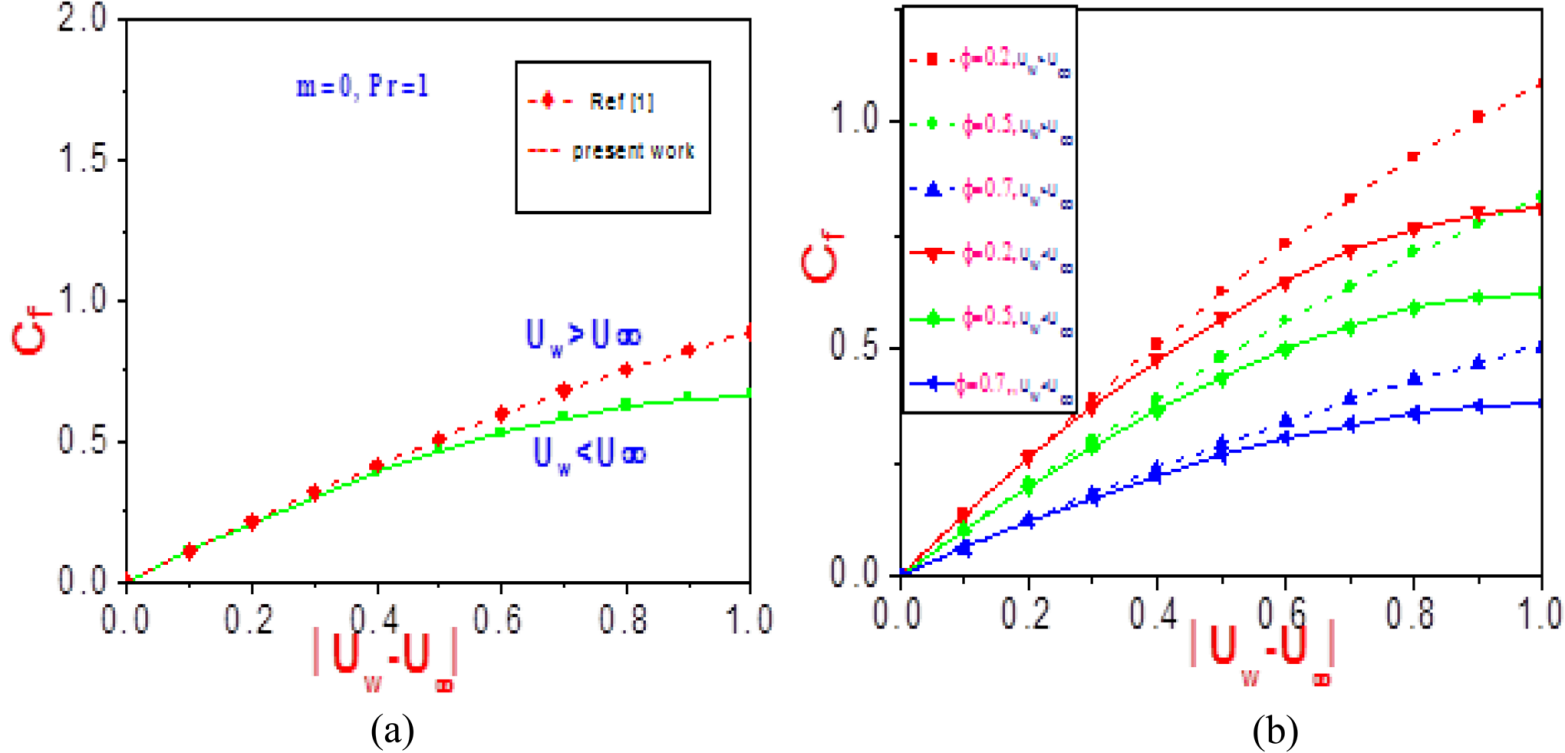
Profiles of shear stress versus normalized velocity difference (**a**) at different values of $\phi$ near the wall $(\eta = 0)$(**b**) for $n = 1$ and without nanoparticles^1^.

**Figure 19:**
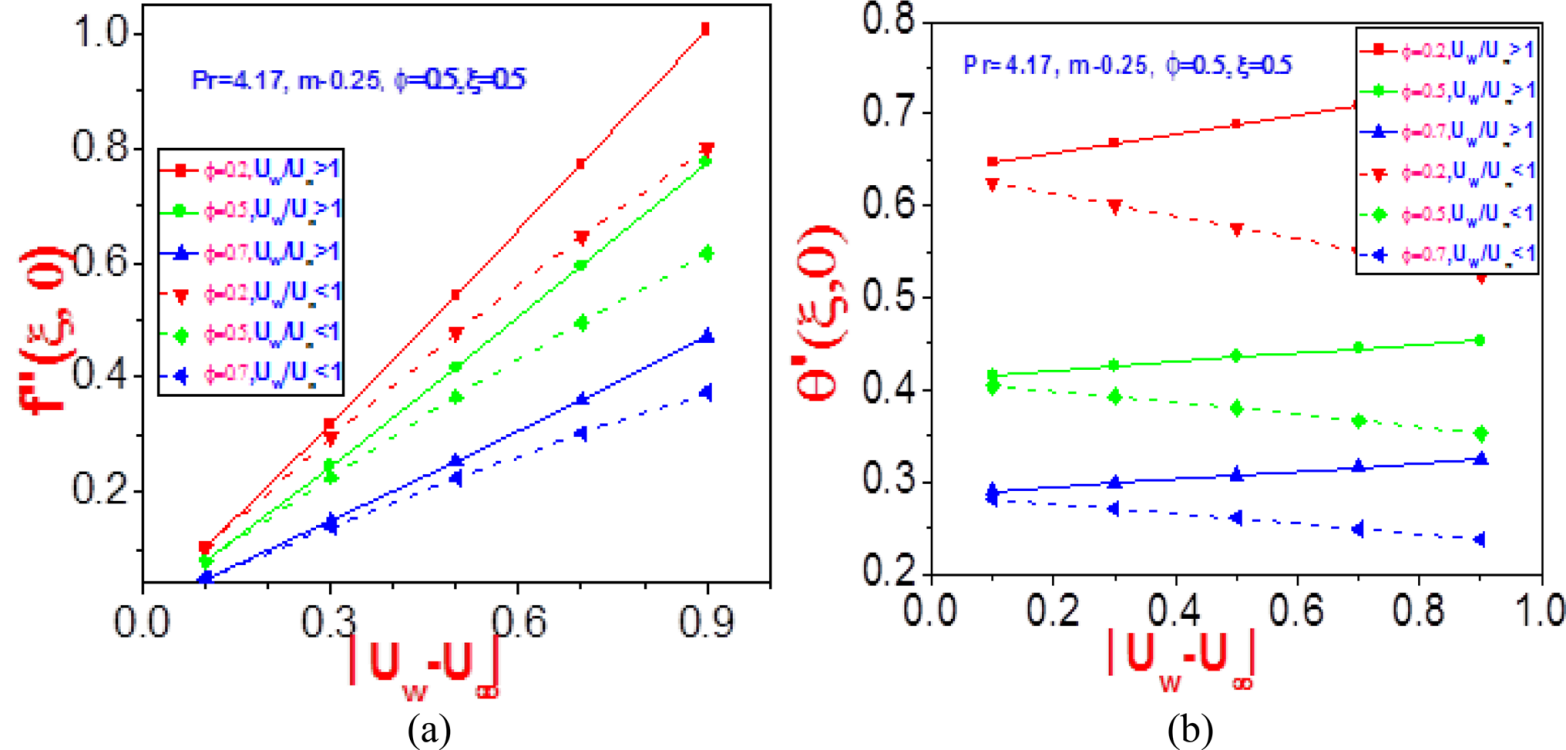
Profiles of (**a**) missing velocity slope $f^{\prime\prime}(\xi ,0)$ and (**b**) missing temperature slope $\theta^{\prime}(\xi ,0)$ with different values of $\phi$ near wall $(\eta = 0)$.

**Figure 20:**
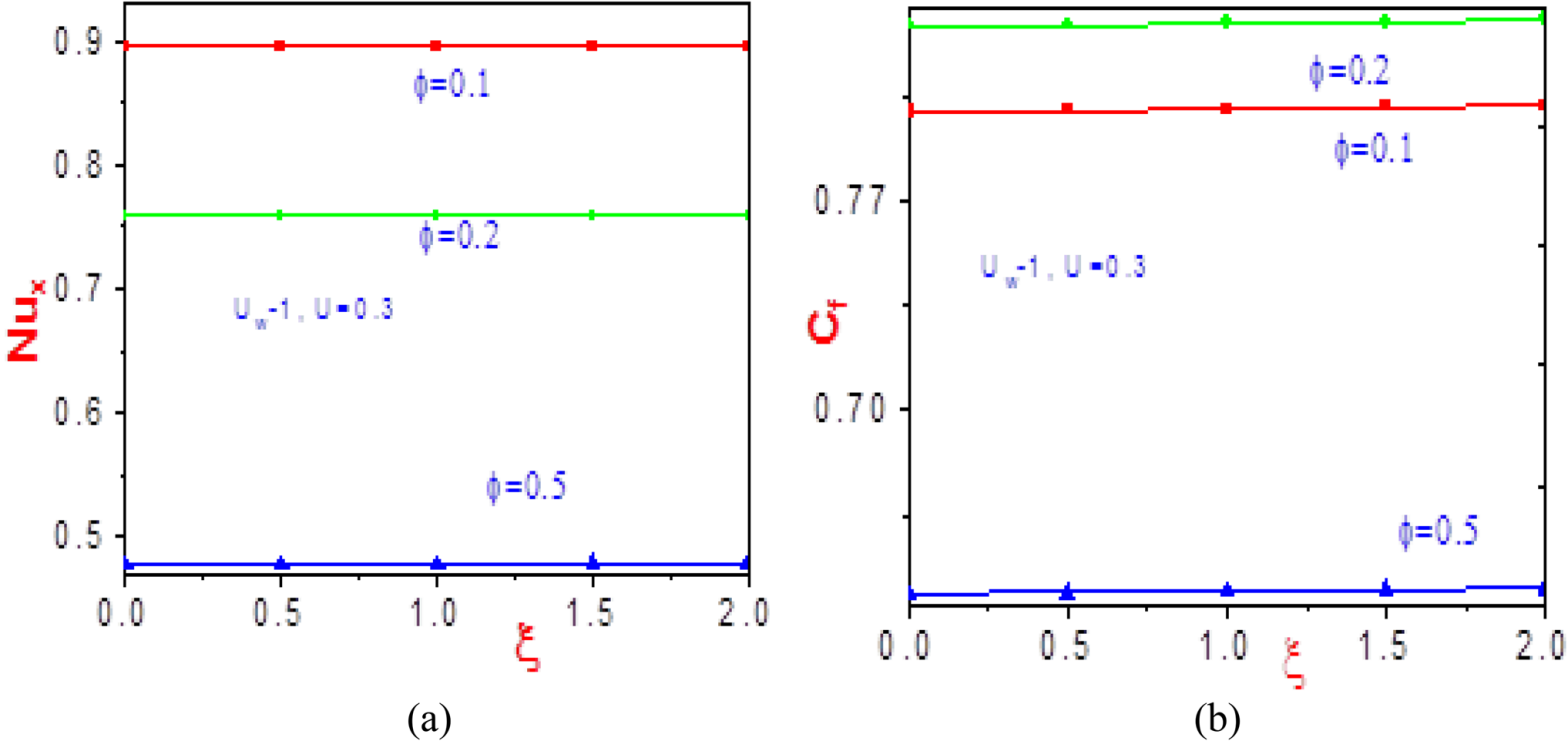
Profiles of (**a**) Nusselt number and (b) shearing stress as a function of nonsimilar parameter $(\xi )$ near the wall $(\eta = 0)$ with different values of $\phi$.

The quantities of technology interest (shear stress and Nusselt number) are presented in Figs. 17, 18, 19 and 20 for different physical terms. Corresponding to the enacted stress, shear stress causes non-Newtonian nanomaterial deformation by slippage in the plane of the stress. Hence, a significant effect of this is noticed in the figures. Additionally, at the fluid boundary, the ratio of heat transfer convectively to conductivity defined the Nusselt number. The heat transfer component at the wall has a strong influence on the various nanomaterials, as depicted in the plots. The figures show the plot for variation in the free stream velocity against the normalized velocities.

## Conclusion

The present work is considered a nonsimilarity solution for a forced convection Newtonian fluid steady. The transformed coupled, nonlinear ordinary differential equations (6)–(7) subject to the boundary conditions are solved numerically by using the local nonsimilarity method ***routine***. The system of equations is of the parabolic type and can be solved by several numeric techniques (local nonsimilarity method). The effects of the solid volume fraction $(\phi )$, free stream velocity $(u_{\infty } )$, constant velocity $(u_{w} )$, Prandtl number $(\Pr )$, and wall temperature exponent $(m)$ on the flow field and temperature field are analyzed. The error of choosing the guess values was determined. The results of the numerical analysis lead to the following conclusions:

- Having better impact when Ag nanoparticles are used because of higher thermal conductivity.
- The fluid flow and temperature field are significantly dependent upon constant velocity and free stream velocity.
- Increasing the values of free stream velocity, the boundary layers for velocity, temperature, missing velocity slope, missing temperature slope are increasing but after a point of separation missing temperature slope is decreasing.
- The normalized velocity difference |${{U_w} - {U_\infty }}$| and nonsimilarity variable *ξ* are established to be the most effective effects on the Nusselt number and shearing stress.

Hence, in engineering and science, the applications of the present investigation cannot be overemphasized. As such, the extension of the study is encouraged to include viscoplastic fluid material in a concentric cylinder is suggested.

## List of symbols

$$\psi (x,y)$$: Stream function
*f*(*ξ*,* η*): Dimensionless velocity
*θ*(*ξ*,* η*): Dimensionless temperature
$k$: Thermal conductivity (W/mK)
$\phi$: Solid volume fraction
$\rho_{nf}$: The fluid density (kg/m^3^)
$(\rho c_{p} )_{nf}$: The heat capacitance of the nanofluid (J/kg K)
$k_{nf}$: Thermal conductivity of the nanofluid (W/mK)
$$k_{nf} /(\rho c_{p} )_{nf}$$: Thermal diffusivity
$\nu_{nf}$: Kinematic viscosity of the nanofluid. (m^2^ s^−^^1^)
$\mu_{nf}$: Dynamic viscosity (kg m^−^^1^ s^−^^1^)
$$\Pr$$: Prandtl number
$${\text{Re}}$$: Reynolds number
$$T$$: Temperature (K)
$T_{w}$: Temperature at fluid sheet interface (K)
$T_{\infty }$: Temperature at free stream (K)
$u$: Fluid velocity component in the x-direction (m/s)
$$u_{r}$$: Reference velocity (m/s)
$u_{w}$: Velocity of continuous sheet (m/s)
$$u_{r}$$: Reference velocity (m/s)
$u_{\infty }$: Free stream velocity (m/s)
$U_{w}$: Ormalized velocity of sheet (m/s)
$U_{\infty }$: Normalized free stream velocity (m/s)
$v$: Fluid velocity component in the y-direction (m/s)
$x$: Stream wise coordinate (m)
$y$: Cross-stream coordinate (m)
$m$: Wall temperature exponent
$C_{f}$: Skin friction factor
$$Nu$$: Nusselt number
